# A bargaining experiment on heterogeneity and side deals in climate negotiations

**DOI:** 10.1007/s10584-017-1975-3

**Published:** 2017-05-04

**Authors:** Greer Gosnell, Alessandro Tavoni

**Affiliations:** Grantham Research Institute, London School of Economics and Political Science, Houghton Street, London, WC2A 2AE UK

## Abstract

The recent global climate change agreement in Paris leaves a wide gap between pledged and requisite emissions reductions in keeping with the commonly accepted 2 °C target. A recent strand of theoretical and experimental evidence establishes pessimistic predictions concerning the ability of comprehensive global environmental agreements to improve upon the business-as-usual trajectory. We introduce an economic experiment focusing on the dynamics of the negotiation process by observing subjects’ behavior in a Nash bargaining game. Throughout repeated rounds, heterogeneous players bargain over the allocation of a fixed amount of profit-generating emissions with significant losses attached to prolonged failure to reach agreement. We find that the existence of side agreements that constrain individual demands among a subset of like countries does not ensure success; however, such side agreements reduce the demands of high-emission parties. Our results highlight the importance of strong signals among high emitters in reaching agreement to shoulder a collective emission reduction target.

**Electronic supplementary material:**

The online version of this article (doi:10.1007/s10584-017-1975-3) contains supplementary material, which is available to authorized users.

Recent developments in climate policy have reaffirmed the perceived importance of minilateral agreements among a small number of countries prior to engaging in large *fora* such as the annual Conferences of the Parties (COPs). A growing literature in political science points to the merits and drawbacks of entering into negotiations among small-n clubs (Keohane and Victor, 2011; Ostrom, 2010; Victor, 2006). At the two ends of the spectrum, one finds bilateral negotiations and almost universal groupings like the United Nations Framework Convention on Climate Change COPs. Most experts agree that bottom-up and top-down approaches are not mutually exclusive (Barrett, 2010; Falkner et al., 2010). Indeed, it appears that some countries have resorted to bilateral deals as a stimulus for action by less motivated countries in global negotiations, a common reading of the USA-China joint announcement to reduce emissions that took place ahead of the 21st COP in Paris. The pledges in the announcement were cemented in the countries’ Intended Nationally Determined Contributions (INDCs) (INDCs as communicated by parties, n.d.).

Would countries commit to emissions cuts if assured of others’ intentions to invest in climate change mitigation? This question is of course an empirical one, and its answer hinges on the success of ongoing international climate negotiations and the ensuing burden-sharing settlement. However, it will take years before the implications of such agreements can be (imprecisely) quantified in terms of emissions reductions. In the meantime, one may approach the issue with other tools, such as theoretical modeling and laboratory experimentation. Inspired by a bargaining model that aims to capture some of the stylized tradeoffs inherent in climate change negotiations (Smead et al., 2014), we introduce a novel economic experiment that focuses on the role of side deals reached by a subset of negotiators in shaping subsequent global negotiations.

Smead and coauthors (Smead et al., 2014) use an agent-based model with learning dynamics to examine past failures and future prospects for an international climate agreement. In the model, agents play an N-player Nash bargaining game (Nash, 1950; Kalai and Smorodinsky, 1975; Muthoo, 1999), where each player’s strategy set is the interval (0,1) representing the range of possible reductions: 1 constituting business-as-usual (BAU) and 0 constituting a complete reduction to zero emissions. In addition to imposing learning dynamics, they modify the Nash bargaining game by introducing an exogenous global emissions target *T* in the interval (0,1). Players maintain the full amount demanded from the global “emissions pie”—where a higher share translates to a higher payoff—only if the sum of all individual demands does not exceed the targeted proportion of BAU emissions (and receive a small fraction *δ* of their demands otherwise)*.* The authors vary a number of parameters in the model and find that player heterogeneity increases the likelihood of success and that prior minilateral agreements can facilitate collective agreement (especially those made among a large number of small players as opposed to a small number of large players, ceteris paribus).

We explore this issue of negotiating on costly emissions reductions in the laboratory. The experimental literature on the avoidance of dangerous climate change has thus far focused on the provision of threshold public goods (Barrett and Dannenberg, 2012; Barrett and Dannenberg, 2013; Dannenberg et al., 2015; Hasson et al., 2010; Milinski et al., 2008; Tavoni et al., 2011). The underlying idea is that, in order to stay within a safe operating space and avoid probabilistic losses, players must invest sufficient resources into a public account (Lenton et al., 2008; Pacheco et al., 2014; Santos and Pacheco, 2011; Steffen et al., 2015; Tavoni, 2013; Vasconcelos et al., 2013). One can view this public good as a minimum collective expenditure in climate change mitigation that ensures staying below an agreed temperature change, such as the often-mentioned 2 °C target.

Since climate negotiations entail agreement on emissions reductions with a view toward remaining within a given threshold, we instead frame the costly mitigation problem as a modified Nash bargaining game. This approach has thus far been neglected in the experimental literature on climate change cooperation. In the game, payoffs accrue only if the groups’ demands fall within a given threshold of available emissions. Negotiators must divide the burden of reducing the size of the emissions pie by agreeing on sufficiently ambitious reductions relative to BAU, which in the game is represented by players’ initial endowments. The underlying assumption is that emissions map one-to-one with wealth. While this assumption is undoubtedly a strong simplification of complex dynamics, it allows us to isolate important features of climate change negotiations, such as the tension between a country’s incentive to keep the largest possible fraction of its emissions and the need to make concessions if the collective target is to be met. That is, future emissions reductions generally bear significant opportunity costs in terms of burdens associated with compliance. Since historical responsibilities are not explicitly modeled, the correlation simply aims to capture the pervasive notion of economic sacrifice on the part of countries that commit to future emissions reductions.

In addition to the experimental methodology employed, we depart from (Smead et al., 2014) in two noteworthy ways. First, in our design, the loss incurred by a group that fails to reach agreement is independent of individual demands. This feature is consistent with the standard bargaining game formulation, which prescribes that out-of-equilibrium payoffs are constant. More importantly, to capture the realistic feature that delay in reaching agreement over ambitious emissions reductions will result in the need to agree on even more ambitious targets in the future, we designed the game to comprise multiple rounds with increasingly stringent targets (see Fig. 1). Hence, while selfish motives still push in the direction of high demands in the hope that others will lead the effort, there is a critical urgency for the negotiating group to meet its target.

**Fig. 1:**
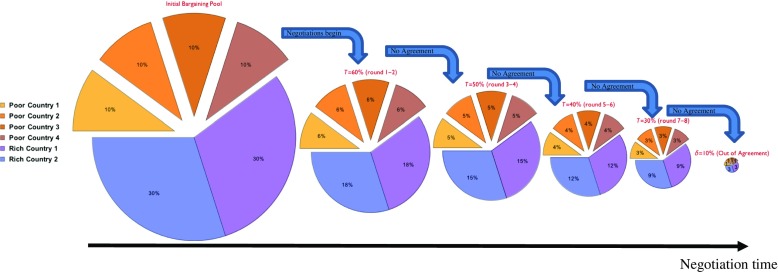
Timing and dynamics of the game. The six-player bargaining game begins with a collective “pie” of £100, which is split between two Rich Countries (each endowed with 30% of the pie, i.e., £30) and four Poor Countries (each endowed with 10% of the pie, i.e., £10). Starting from this initial allocation of wealth/emissions, the group faces sequential rounds of bargaining on progressively tighter targets. The figure depicts the wealth/emissions distribution ensuing from each target if Countries were to reduce symmetrically

Strategic implications of costly haggling, i.e., costs associated with delay in reaching agreement, have been studied extensively. The alternating-offers model entails the partition of a cake between a proposer and a second mover (Rubinstein, 1982). If the latter rejects the offer, she becomes the proposer and the process is repeated. This alternation of roles continues until an agreement is struck, at which point the cake is divided accordingly. The game-theoretic solution predicts instantaneous agreement on the division of the cake, with the proposer securing a weakly larger share, depending on the discount factor. The game analyzed here differs along the following dimensions: number of players (we focus on multilateral bargaining), timing of the proposals (negotiators move simultaneously), horizon (players have a finite number of rounds to reach an agreement), and disagreement costs. In the alternating-offers model, costs of inaction arise with the first rejection and can be thought of as (partial) spoiling of the cake: in the limit, if both players perpetually disagree, their payoffs vanish. Here, the losses are not smooth over time, as is evident from Fig. 1. Furthermore, players do not bargain on the status quo, as the climate change problem requires agreement on shrinking the cake from the outset.

Our bargaining game also relates to the ultimatum game, the simplest form of the alternating-offers model where only the final two stages are considered. Hence the ultimatum: the responder’s choice is again confined to acceptance or rejection of the offer, with rejection implying a 0 payoff for both players. Under complete information, the subgame perfect Nash equilibrium involves a rational self-interested proposer offering nothing (or an arbitrarily small share) and the responder accepting. However, nontrivial offers have been consistently found in experimental settings due to the proposers’ concerns for fairness and fear of rejection of offers below an acceptable threshold (Güth et al., 1982; Forsythe et al., 1994). In common with the above, our game centers upon issues of burden sharing that are likely to trigger fairness considerations. However, the multilateral and simultaneous nature of the repeated negotiations we simulate in the lab—coupled with the introduction of a target requiring coordination—introduces additional considerations, such as group-level efficiency and reputation. We further explain the implications of the game’s design features and discuss its equilibria as well as its relation to the experimental literature in parts (a) and (b) of the Supplementary Information (SI).

## Methods

In the experiment, groups of six “Countries” negotiate over a maximum of eight rounds on increasingly ambitious collective emissions reduction targets. In each round of negotiation, Countries individually demand to keep a proportion of their endowed (BAU) emissions with the shared group goal of shrinking the global pie in accordance with the exogenous global reduction target.

Each treatment consists of up to eight rounds of a Nash bargaining game framed as a climate change negotiation, where the negotiation terminates if the group meets the prescribed Global Target *T* in a given round. The Global Target becomes more difficult to attain as the game progresses, beginning at *T* = 60% of global wealth and reducing by 10% every two rounds (i.e., *T* = 50% in Rounds 3–4, *T* = 40% in Rounds 5–6, and *T* = 30% in Rounds 7–8). If the group does not meet the target by the end of Round 8, negotiation terminates and group members each receive *δ* = 10% of their initial endowment (regardless of their demands in the final round) as an unavoidable consequence of “dangerous” climate change.

In every round, group members—each acting as a delegate representing one Country in the negotiation—engage in what we term the Global Negotiation stage. In this stage, each delegate demands to keep a proportion of her Country’s endowed emissions, which is perfectly correlated with its wealth in the game. If the group’s aggregate demand does not exceed the corresponding Global Target in a given round, the target is met and each subject in the group receives the proportion she demanded in that round. If the target is not met, there is no payout for the round and negotiations continue to the next round.

We implement five variants of the bargaining experiment: Symmetric (SYM), Asymmetric (ASYM), Poor Side Deals (PSD), Rich Side Deals (RSD), and All Side Deals (ASD).^1^ All groups’ aggregate monetary endowments are £100 (approximately US$156). In treatment SYM, all Countries begin with a symmetric endowment of £16.67. All other treatments are characterized by asymmetry in the distribution of endowments (and corresponding impact on global emissions). In these treatments, four Poor Country delegates each receive an endowment of £10 and two Rich Country delegates each receive an endowment of £30 (see Table 1).

**Table 1:** Game design

	SYM	ASYM	PSD	RSD	ASD
Endowments	All (×6): £16.67	Poor (×4): £10 Rich (×2): £30	Poor (×4): £10 Rich (×2): £30	Poor (×4): £10 Rich (×2): £30	Poor (×4): £10 Rich (×2): £30
Side Deals	None	None	Poor	Rich	Poor Rich

All treatment conditions consist of eight rounds of negotiation. Treatments without Side Deals—SYM and ASYM—feature only single-stage rounds, as depicted in Fig. 1. In each of these rounds, delegates independently and simultaneously decide on individual (i.e., Country-level) demands. The software computes the aggregated “global” demand of the group and displays both global and individual demands in a subsequent screen in absolute and percentage terms.

In treatments containing Side Deals (PSD, RSD, and ASD), either one or two subsets of delegates—belonging to the same wealth/emissions category, i.e., Poor, Rich, or Poor and Rich, respectively—may collectively place binding constraints on own individual demands in the two upcoming Global Negotiation stages.^2^ Accordingly, these Side Deals take place prior to the Global Negotiation stages of Rounds 1, 3, 5, and 7. The outcome of a Side Deal—the *Agreed Maximum Demand*—applies only to Countries who took part in the Side Deal, though it is revealed to all Countries within the group prior to the subsequent Global Negotiation stages. The Agreed Maximum Demand is the mean of the *Maximum Demands*, i.e., the answers of the Side Deal participants to the following question (in the PSD treatment): “What is the maximum percentage of emissions/wealth that you think is appropriate for each Poor Country to demand in each of the two upcoming global negotiations?”

To be clear, we provide the following hypothetical example of Side Deal implementation in the PSD treatment. Prior to the Global Negotiation stage of Round 1, all four Poor Countries will determine an Agreed Maximum Demand, which is a binding constraint on the Poor Countries’ individual demands in the Global Negotiation stages of Rounds 1 and 2. In this Side Deal stage, if two Poor Countries choose a Maximum Demand of 80 and two choose a Maximum Demand of 60, the resulting Agreed Maximum Demand is (2 * 60 + 2 * 80) / 4 = 70. Poor Countries may then only individually demand to keep up to 70% of their own initial endowment in the Global Negotiation stages of Rounds 1 and 2. If the group collectively fails to reach the Global Target of 60% of global wealth/emissions by the end of Round 2, Poor Countries will again enter a Side Deal stage and similarly determine a new Agreed Maximum Demand that pertains to the Global Negotiation stages of Rounds 3 and 4—when the Global Target is reduced to 50%—and so on.^3^

## Results

### Global success

#### Asymmetry and side deals

Table 2 provides a descriptive overview of group performance dynamics across treatments. First, we see that *all* symmetric groups had reached agreement by the end of the fourth round of negotiations. When comparing success rates within the first four rounds, the SYM groups outperform the ASYM (proportion test, *p* = 0.101, *z* = 1.64), RSD (proportion test, *p* = 0.049, *z* = 1.96), and ASD (proportion test, *p* = 0.062, *z* = 1.86) groups. This finding is in contrast to the results in (Smead et al., 2014), where the authors find that asymmetry of endowments increases the likelihood of agreement. A second, more relevant finding is the limited impact of Side Deals on negotiation outcomes. When comparing ASYM groups to all groups containing Side Deals (both pairwise and combined), we do not find conclusive evidence that treatments containing Side Deals improve upon global negotiations that occur among asymmetric actors in the absence of Side Deals, in terms of both agreement velocity and (individual- and group-level) demands. Thus, human behavior in a laboratory setting modeled closely after (Smead et al., 2014) does not appear to corroborate the simulation data of their agent-based model.^4^

**Table 2:** Success rate by target level

	Rounds 1–2	Rounds 1–4	Rounds 1–6	Rounds 1–8
SYM	63.6%	100.0%	100.0%	100.0%
ASYM	64.3%	78.6%	85.7%	85.7%
PSD	80.0%	80.0%	90.0%	90.0%
RSD	50.0%	70.0%	90.0%	100.0%
ASD	54.5%	72.7%	90.9%	100.0%

Percentages indicate the proportion of groups in each treatment who had reached agreement by a given threshold round

However, we do find evidence that Side Deals among Rich Countries are significantly more binding in “successful” groups—which we define to be those groups who reached agreement without any efficiency losses (i.e., in Rounds 1 and 2)—than in unsuccessful groups. Considering groups who participated in either the PSD or ASD treatments, the Agreed Maximum Demands of the Poor do not significantly differ across successful and unsuccessful groups. However, if we look at groups in either the RSD or ASD treatments, the Agreed Maximum Demand of the Rich significantly differs across successful and unsuccessful groups (Wilcoxon-Mann-Whitney test, 62.3 in successful groups vs. 72.6 in unsuccessful groups, *p* = 0.028, *z* = 2.193). In fact, these differences hold—albeit with reduced statistical power—if we compare these groups within RSD (WMW, 58.4 vs. 66.6, *p* = 0.106, *z* = 1.616) and within ASD (WMW, 65.5 vs. 78.6, *p* = .067, *z* = 1.830) separately. This result indicates that the extent to which high-emission countries tie their hands is of paramount importance for group success, though the same does not hold for low-emission countries.

#### Unconditional cooperation

We can also examine the effect of group composition on negotiation success in terms of proportion of individuals inclined to cooperate unconditionally, where “unconditional cooperators” are those who demand at most a percentage equivalent to the Global Target (*T* = 60%) in Round 1. Pooling all treatments together, we find that there is almost exactly one additional unconditional cooperator on average in successful groups, as compared to unsuccessful groups (WMW, 3.89 vs. 2.86, *p* = 0.003, *z* = −2.945). This result remains intact when we exclude SYM from the comparison (WMW, 3.821 vs. 2.647, *p* = 0.007, *z* = −2.703).

We further investigate the importance of Rich versus Poor cooperation and find that successful groups have almost double the number of Rich unconditional cooperators as unsuccessful groups, on average (WMW, 1.679 vs. 0.882, *p* = 0.001, *z* = −3.426), while successful groups and unsuccessful groups are not significantly different in terms of the number of Poor unconditional cooperators (WMW, 2.14 vs. 1.76, *p* = 0.400, *z* = −0.842). Taken together, these results reinforce the notion that strong commitment and unconditional cooperation by Rich Countries hold paramount influence in determining the success of multilateral negotiations.

### Individual demands

#### Wealth redistribution

**Fig. 2:**
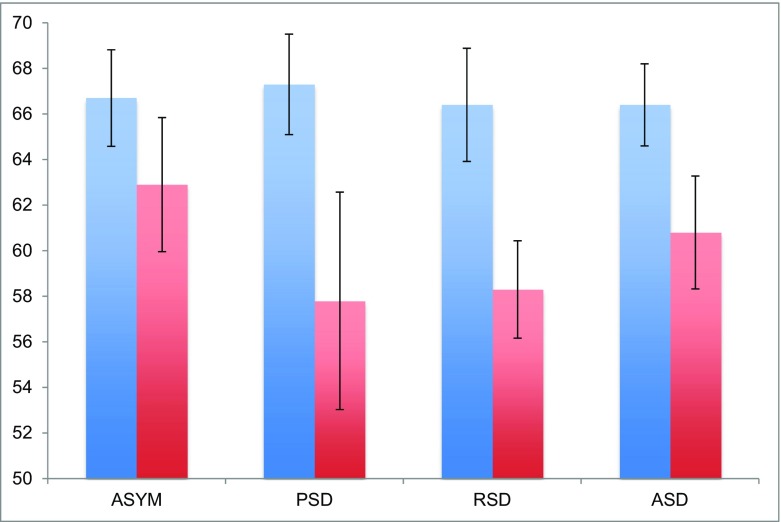
Average initial demands (and standard error bars) by Poor (*blue*) and Rich (*red*) negotiators in treatments with asymmetric endowments

An interesting question pertains to the behavior of the two different player types in the asymmetric treatments: is there evidence of redistribution from the Rich to the Poor, in the form of lower demands by the wealthy? In asymmetric groups, we find evidence of such redistribution: the Poor demand 66.7% of initial wealth and the Rich demand 60.2% in the first round (i.e., across all groups in the sample), on average (WMW, *p* = 0.000, *z* = 3.381). More interesting is the apparent dependence of this disparity on whether Side Deals take place prior to the first global negotiation stage. Comparing the average initial demands of Poor and Rich Countries within treatment groups (Fig. 2), we see substantial differences under PSD (WMW, 67.3 for Poor vs. 57.8 for Rich, *p* = 0.071, *z* = 1.805), RSD (WMW, 66.4 for Poor vs. 58.3 for Rich, *p* = 0.031, *z* = 2.154), and ASD (WMW, 66.4 for Poor vs. 60.8 for Rich, *p* = 0.092, *z* = 1.686), though this difference is attenuated in ASYM (WMW, 66.7 for Poor vs. 62.9 for Rich, *p* = 0.240, *z* = 1.186). Consistent with (Tavoni et al., 2011), it thus appears that Side Deals increase the salience of the inequality, inciting fairness motivations that are manifested through a downward shift in Rich Countries’ demands.

**Fig. 3:**
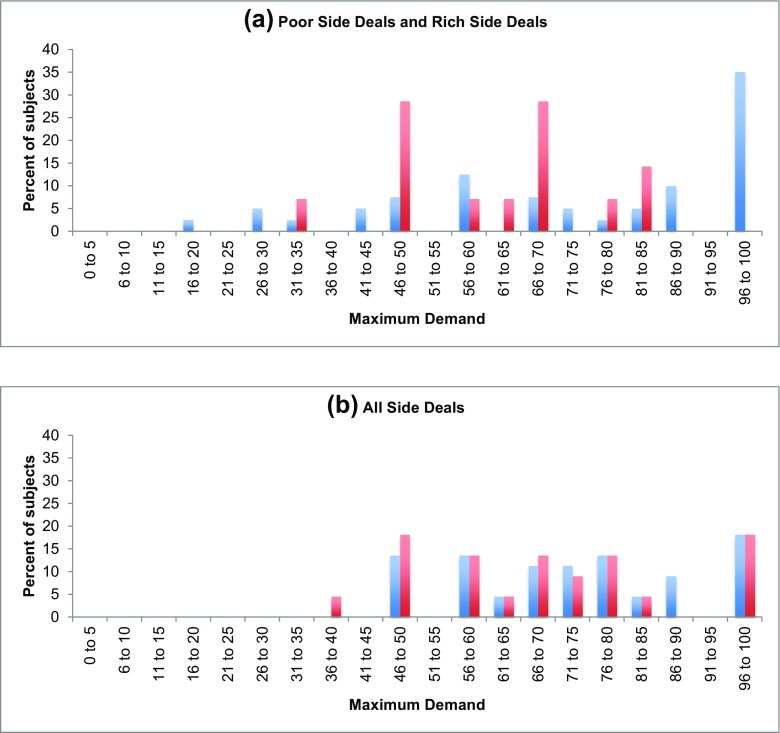
Distributions of Maximum Demands by Poor (*blue*) and Rich (*red*) players in treatments with **a** Poor or Rich Side Deals (PSD or RSD) and **b** both Poor and Rich Side Deals (ASD). Since only Poor (Rich) Countries input Maximum Demands in the Poor (Rich) Side Deals treatment, **a** combines the data from these two treatments for ease of comparison

This increased salience is especially apparent when the Side Deals pertain to only one subgroup (i.e., *either* the Poor *or* the Rich), as evidenced by the Side Deal inputs (i.e., Maximum Demands) chosen by Poor and Rich negotiators in the various treatments containing Side Deals (see Fig. 3). For instance, in PSD, the modal Maximum Demand input in the Side Deal pertaining to the first two rounds of Global Negotiation is 100%, and a vast majority of Poor Countries chooses values at or above the Global Target of 60%. On the contrary, in RSD, not a single player chooses a preferred Maximum Demand of 100%, and a majority of Rich Countries selects a value in the range of 50–70%. However, when both Poor and Rich Countries engage in Side Deals, the distribution of Maximum Demands between the two player types is strikingly similar. Hence, negotiators’ decisions are clearly shaped by the initial conditions and institutional frameworks surrounding the bargaining process.

#### Conditional demands

We additionally explore whether other group members’ demands are an important determinant of individual decisions. Indeed, we find evidence of “carbon leakage” across country types; that is, we find a significant positive effect of past cooperation by the Rich (Poor) on Poor (Rich) Countries’ demands (Table 3). Specifically, Poor Countries increase their average demand in the present round by almost four percentage points for every additional Rich Country that cooperated (by demanding a percentage less than or equal to the target) in the previous round. Similarly, Rich Countries increase their demands by almost three percentage points for each additional Poor Country that cooperated in the prior round. We do not find evidence that Countries take advantage of the cooperation of like Countries.

**Table 3:** Conditional demands of Poor and Rich

	Poor demand	Rich demand
Rich cooperated	3.865** (1.768)	1.694 (2.540)
Poor cooperated	−0.020 (1.047)	2.685*** (0.813)
Constant	59.401*** (6.194)	53.175*** (3.578)
Groups	26	26
Subjects	104	52
Obs	356	178
Controls	Yes	Yes

The dependent variable in this regression indicates the individual demands over the course of negotiation. The independent variables represent the number of Rich and Poor Country representatives (respectively) who cooperated in the prior round by demanding less than or equal to the Global Target. Controls include gender, Annex 1 nationality, stated primary motivation, Global Target, and the difference between the group demand and the target in the prior round of negotiations. There are 26 groups in heterogeneous treatments that negotiated past the first period, and these are the groups considered here. Robust errors are clustered at the group level. Standard errors are reported below estimates in parentheses

**p < 0.05, ***p < 0.001

## Discussion

We explore the impact of country heterogeneity and minilateral agreements on climate bargaining processes in a controlled laboratory setting. Our findings stress the importance of early unconditional cooperation by high emitters in efficiently allocating emissions reductions consistent with a global reduction target. However, the experimental data also suggest that some degree of carbon leakage may take place, in the sense that ambitious commitments from high emitters may reduce the abatement efforts of low emitters. That is, we find evidence that the two player types tend to take advantage of the other type’s cooperation, demanding to keep a proportion of emissions closer to their BAU as the other type’s concessions increase.

We do not find that “tying your hands” ahead of the inclusive negotiations necessarily promotes cooperation, although Side Deals among various subsets of players do affect bargaining dynamics. Importantly, under conditions of heterogeneity, the disparity between the average demands of the two negotiator types widens in the presence of Side Deals, suggesting an even larger role for high-emission (i.e., industrialized or newly industrializing) countries.

What are the implications for international climate negotiations going forward? In light of the vast heterogeneity across countries in terms of both wealth and emissions, the above findings suggest that the infrastructure around which climate change negotiations revolve are crucial determinants of process dynamics. Specifically, our results indicate that low-emission countries will not increase their ambitions in the near term as a result of side agreements by high-emission states, such as the joint announcement made by China and the USA late in 2014. Therefore, high-emission countries will likely need to commit to still further reductions to maintain a current trajectory consistent with limiting mean global temperature rise to 2 °C (Friedlingstein et al., 2014). Furthermore, given the strong initial commitments by high emitters necessary to ensure success, the tendency to free ride off of unlike countries means that (generally poor) low-emitting countries—so long as they remain as such—are unlikely to increase their ambitions over time. A prompt and effective agreement thus hinges on strong, unconditional commitments by industrialized and newly industrializing countries, a condition that led to strong contention under the framework of the Kyoto Protocol.

Notwithstanding the recent non-binding global agreement at COP 21 in Paris—which depends on future negotiations to close the gap between INDC pledges and the requisite emissions reductions to keep with the 2 °C threshold—the above conclusions cast a shadow on the prospects for a sufficiently ambitious outcome of ongoing global climate negotiations. Our results indicate that minilateral agreements are not “game changers,” at least not without significantly ambitious reduction commitments by high-emission countries, which thus far have not materialized. To make matters worse, while the game analyzed here brings potentially disruptive wealth and responsibility heterogeneities to center stage, un-modeled obstacles further hinder climate change cooperation. For instance, the game equates current emissions with responsibilities, neglecting historical accountability and future development requirements. Moreover, only six negotiators must strike an agreement, which simplifies the coordination problem faced by the 197 parties to the UNFCCC.

Importantly, negotiators outside the lab have to rely mostly on voluntary commitments lacking legal force, as demonstrated by the shift from legally binding emission targets to pledge and review mechanisms witnessed in the Paris COP in December 2015. Hence, our voting system for determining the maximum allowable demands in the global negotiations may oversimplify the task of “tying one’s hands” compared to the real negotiations, where processes leading to minilateral agreements may vary and countries face incentives to renege on earlier promises if they stand to gain from doing so. However, committed coalitions may use the threat of diplomatic and economic measures, such as “naming and shaming” and trade sanctions, in order to induce cooperation by less ambitious states. Indeed, there are examples of international agreements without binding rules that were successful despite their voluntary nature and reliance on international scrutiny, such as the Helsinki Declaration on human rights (Bodansky, 2015).

On the other hand, climate negotiations can rely on more instruments than those available to our subjects. Here, there are no direct transfer mechanisms, such as the Adaptation Fund and climate finance. In addition, climate co-benefits may lure countries to join small-n clubs early on, providing much needed leadership (Keohane and Victor, 2011; Ostrom, 2010; Victor, 2006). Our game focuses on short-run costs of mitigation, neglecting such opportunities. Yet, policy tends to be defined by short-term incentives and high discounting, as confirmed by the insufficient ambition of the INDCs pledged prior to COP 21 (INDCs as communicated by parties, n.d.; International Energy Agency, 2015). The implementation of these pledges will likely be further hindered by myopic policymaking (Barrett and Dannenberg, 2016), as well as politicians’ (and their constituents’) differing stakes and perspectives with regard to the magnitude of climate damages (Marchiori et al., 2017). Hence, under the current framework, the global community runs the risk of bargaining toward ineffective agreements in the coming crucial decades. We therefore urge policymakers to consider additional complementary or stand-alone mechanisms to increase the likelihood of avoiding dangerous climate change.

## Electronic supplementary material

ESM 1 (DOCX 562 kb)

## Footnotes

1 A total of 336 subjects participated in 20 experimental sessions. Eleven groups participated in SYM, 14 in ASYM, 10 in PSD, 10 in RSD, and 11 in ASD.

2 Alternatively, given a sufficiently large sample size, the effect of voluntary or non-binding side deals could be elicited by allowing experimental subgroups to vote on whether to implement a given upper bound on demand (e.g., the AMD presented here, or an amount proposed by a group leader); after which, groups who vote in favor become constrained and those who do not remain unconstrained. We elected to average the Maximum Demands in order to convey a sense of ownership over the AMD while maintaining sufficient statistical power to detect meaningful differences in outcomes across treatment groups with and without side deals. See the SI (a) for a discussion of the tradeoffs between external validity and statistical power.

3 See SI for further details, including Screenshots 4–8 for visual representations of the above material as displayed in the experimental instructions.

4 For further analysis and robustness checks, see part (f) of SI.
