# Supplementary material for: A bargaining experiment on heterogeneity and side deals in climate negotiations
Source: Clim Change. 2017 May 4;142(3):575–86. doi: 10.1007/s10584-017-1975-3 (PMC6991966; doi:10.1007/s10584-017-1975-3)
Supplement: Supplementary file 1 — (DOCX 562 kb) [file 10584_2017_1975_MOESM1_ESM.docx]

**Supplementary Information for:**

**A Bargaining Experiment on Heterogeneity and Side Deals in Climate Negotiations**

**Greer Gosnell^[[1]](#footnote-1), 2^, Alessandro Tavoni^[[2]](#footnote-2), a^**

**(a) Experimental design and related literature**

In an effort to construct an experiment that captured important elements of abrupt climate change yet retained the simplicity necessary to ensure internal validity in a laboratory setting, we made several simplifying design decisions. In the main text we mention these decisions in our discussion of how the game relates to (or departs from) the literature. Here we expand on the motivations for and implications of such choices.

While the experimental literature on climate change negotiations tends to center upon public goods games, we depart from this mechanism in several ways for two primary purposes: a) to enhance the relevance of the context, and b) to provide an empirical test of the agent-based model proposed in (1). Rather than employing a voluntary contribution mechanism devoid of context, we narrow our interest to pertain solely to climate change negotiations, where the instructions provide clear background information on the economic complexities associated with this pervasive externality.

For instance, the dynamic nature of the Global Target captures the cost of delaying legislation to curb greenhouse gas emissions, a stock pollutant with long-term atmospheric warming effects. The target persists over two rounds to allow for learning. While any time lapse theoretically increases the necessity of stronger future abatement commitments to reach a given target, the slow and lagged process of climate change and the relative frequency of negotiations allows for fairly stable global goals in the short term, so that learning can occur from one negotiation to the next. Note that this game neglects gradual damages, since we are concerned with the large costs associated with failure to reach a timely agreement on a target. Such targets can be interpreted as either emerging from scientific evidence or from political discourse (for example, 2ºC).

In addition to the detailed description of global climate change provided in the instructions, various features of the game—e.g., the responsibility dichotomy between asymmetric Country types, the termination of negotiation if the target is reached, and the correlation between emissions and wealth—were designed to mimic the climate context as closely as possible while maintaining the simplicity necessary to ensure the game’s comprehensibility. For instance, assigning players to represent either “Rich” (high-emission) or “Poor” (low-emission) Countries mimics the accepted categorization of countries in the COP negotiations, where much diplomatic effort revolves around sharing the “common but differentiated responsibilities” between developed Annex I countries and less developed non-Annex I countries.^[[3]](#footnote-3)^ It additionally captures elements of current emissions responsibility as well as the disproportionate sacrifice associated with deviating from BAU.

Finally, the composition of the groups—where a third of the countries represented are responsible for 60% of global greenhouse gas emissions—is reflective of the 54% for which the top three global players (United States, European Union, and China)—who engaged in pre-COP 21 minilateral discussions—are responsible (2).^[[4]](#footnote-4)^ To ensure that we had sufficient statistical power to detect meaningful differences across treatment groups with and without Side Deals, we did not allow Countries in these treatments to opt out of Side Deals. While in real negotiations it is often possible to avoid public commitments, political pressure may make doing so costly. One could argue, by way of example, that the pressure for China and the U.S. to form an agreement in the run-up to COP 21 together with pending U.S. presidential elections meant that opting out of this “side deal” was not a politically desirable option for either party. Indeed most countries submitted their Intended Nationally Determined Contributions prior to the Paris conference, even if they were not obliged to do so.

**(b) Game equilibria**

As shown in the two propositions contained in the SI to (1), the threshold bargaining game employed here features two types of strict Nash equilibria, which can be either *disagreement outcomes* or *feasible solutions.* In disagreement outcomes, all players are unwilling to make sufficient concessions, i.e. the other five players in one’s group demand too much for any single player to facilitate agreement by reducing her demand (so that the threshold in a given round is unattainable). In feasible solutions, the threshold is exactly met and everyone is better off than in disagreement, i.e. every player’s demand is larger than *δ*=10% (the amount one can get out of agreement).

For the sake of tractability, let us focus on the treatments without side deals, which allows us to bypass the strategic implications of constraining future decisions. As illustrated below, for our parameters, feasible solutions are preferable equilibria in the sense that they Pareto dominate the disagreement outcomes, yet free-riding incentives pull players toward disagreement. In our game, there are four thresholds corresponding to different pairs of rounds—*T*=60%, *T*=50%, *T*=40%, and *T*=30%—so essentially one can treat each pair of rounds as a separate game where bargaining takes place on the relevant *T*. Given the relative values of *δ* and *T* in the game, along with the shrinking of *T* over time, groups maximize their payoffs by coordinating on a feasible solution in the first two rounds. As bargaining continues to later stages, wealth is inevitably lost due to the tightening target and agreement becomes less appealing. Note however, that regardless of the distribution of endowments, players always have an incentive to strike an agreement compared to a disagreement outcome. For example, suppose that the negotiations reached the final round. Failing to strike an agreement would mean a take-home payoff of roughly £1.7 in SYM (and £1 and £3 for Poor and Rich, respectively, in all other treatments). These values are lower than the payoff that players can secure by each demanding to keep 30% of their endowments in Round 8 (i.e. £5 in SYM, £3 and £9 for Poor and Rich, respectively, in all other treatments).

Of course, subjects may deviate from symmetric behavior, perhaps due to the presence of obstinate free-riders. While this matter is an empirical one, here we briefly show that some degree of free riding may be sustained in the game, so long as a sufficient number of players is willing to compensate such behavior. Let us restrict attention to SYM, for simplicity. When *T*=60%, up to three free riders can be tolerated in the sense that if the other three players are willing to shoulder (equally) the entire burden of shrinking the pie by 40%, they will still earn £3.3 each, which is more than they would earn out of agreement. By a similar token, up to two free riders are sustainable when *T*=50% or *T*=40%, and only one free-rider can be sustained when *T*=30%.

**(c) Experimental implementation**

We employ a design that allows for between-subject and between-group analysis. Each subject participated in a group negotiation of up to eight rounds. Once all groups finished the negotiation, subjects were prompted to complete a brief questionnaire to assess motivation, strategic decision-making, and demographic heterogeneity (see section (d) for the experimental instructions, as well as section (e) for the full questionnaire). Additionally, each subject answered a risk preference elicitation question equivalent in structure to the standard question used in (7, 8), with payoffs scaled down to 10% of those used in their experiment. The question asked subjects to select one of five incentive-compatible 50-50 gamble options, where payoffs increase linearly in expected payout and “riskiness” of the gamble, as measured by the standard deviation of the two possible payouts, which ranged from £0.60 to £4.20. The outcome of the gamble was determined individually by a coin toss upon payment for the study.

At the beginning of the experiment, subjects received both written and oral instructions. Each subject had to correctly complete a test for understanding before the experiment began. At the end of the experiment, subjects privately received their experimental earnings in cash, in addition to a £5 show-up fee, totaling £16.80 on average. All experimental decisions were made on a computer screen using the experimental software Z-Tree (9).

A total of 336 student (undergraduate and postgraduate) and non-student subjects volunteered to participate in 20 experimental sessions, most comprising three groups of six subjects (four sessions contained only two groups). The experiment took place at the London School of Economics (LSE), though experimental participation is not restricted to LSE students. In our sample, 50.9% of subjects are female, 42.3% are from Annex I countries (and 52.6% are from countries that engaged in “side deals” prior to COP 21: 5.2% USA, 36.3% EU, and 11.1% China), 47.6% are undergraduate students, and 33.6% are graduate students. The average age of our subjects is 23.5 years (SD=5.99). Student participants come from various disciplines (10.4% Business; 14.9% International Policy, Law, or Government; 8.0% Geography & Environment, 13.1% Economics).

**(d) Experimental instructions for participants of the ASD treatment**

Welcome to the experiment! In this experiment, you can earn money. In addition to your earnings from the experiment, you will receive a £5 show-up fee. During the course of the experiment, please do not talk to other participants. We will now read the experimental instructions aloud. Once we have finished reading, raise your hand if you have questions and we will be with you shortly to answer them. At the end of Part A of the instructions you will find some questions that are meant to ensure that you understand the rules of the experiment. Please answer all questions and signal us by raising your hand when you have finished, so that we may check your answers.

***Background: Climate change*.** Climate change is viewed as a serious global environmental problem. The vast majority of climate scientists expects the global average temperature to rise by 1.1-6.4°C before 2100, where a rise of 2°C is generally considered to be dangerous climate change. There is hardly any disagreement that mankind largely contributes to climate change by emitting greenhouse gases, especially carbon dioxide (CO_2_). CO_2_ originates from the burning of fossil fuels such as coal, oil, or natural gas in industrial processes and energy production, as well as from combustion engines of cars and lorries. CO_2_ is a *global* pollutant—that is, each unit of CO_2_ emitted has the same effect on the climate regardless of the location where the emissions occur. Dangerous climate change will result in significant global costs, which get worse over time if agreement is not reached. International climate change negotiations involve yearly meetings where delegations representing different *countries try to strike a global agreement on emissions reductions* that are consistent with the goal of avoiding dangerous climate change. Here you will be asked to negotiate such costly emissions reductions on behalf of the Country to which you will be assigned. Your choices, together with those of the other ‘Countries’, will determine your payout from the experiment.

***Rules of play*.** Now we will introduce you to a game simulating international climate change negotiations. In total, six Countries are involved in the global negotiation. That is, in addition to you, there are five other negotiators in your negotiation group, and each of you represents one Country. The six Countries account for *all* global wealth and CO_2_ emissions (for simplicity, we disregard other greenhouse gases in the experiment). While excessive emissions impose global costs, individual Countries rely on productive processes which create emissions in order to generate wealth: for every 1 billion tons of CO_2_ ‘emitted’ in the game, you receive £1. Hence, reducing emissions is costly. Your decisions in the experiment are anonymous. To guarantee anonymity, you will be randomly assigned to one type of Country (Rich or Poor), and you will be identified by one of the following names: Rich Country 1, Rich Country 2, Poor Country 1, Poor Country 2, Poor Country 3, Poor Country 4. Your name will appear on the lower left side of your screen once the experiment begins. At the beginning of the experiment, you will receive a sum of money that represents your Country’s wealth. This wealth mirrors your Country’s CO_2_ emissions. Therefore, throughout the instructions and the experiment, we will refer to wealth and emissions interchangeably. The current situation in your negotiation group can be summarised as follows:

- **Two Rich Countries** each emit 30 billion tons of CO_2_ and earn **£30** in doing so;
- **Four Poor Countries** each emit 10 billion tons of CO_2_ and earn **£10** in doing so;
- The resulting Global Emissions amount to **100 billion tons of CO_2_** (2×30 billion tons of CO_2_ + 4×10 billion tons of CO_2_)
- Hence, **Global Wealth** is equal to **£100** (2×£30 + 4×£10)

Due to the threat of dangerous climate change, the goal is to agree on an aggregate level of Global Emissions that does not exceed a given Global Target. In the following experiment, you will participate in up to 8 rounds of climate change negotiations, where the global costs from *not* reaching agreement increase every 2 rounds. **Accordingly, the Global Target decreases every two rounds, as follows:**

- Rounds 1-2: **60% of current emissions** (60 billion tons of CO_2_)
- Rounds 3-4: **50% of current emissions** (50 billion tons of CO_2_)
- Rounds 5-6: **40% of current emissions** (40 billion tons of CO_2_)
- Rounds 7-8: **30% of current emissions** (30 billion tons of CO_2_)

To be clear, since current global emissions are 100 billion tons of CO_2_, an agreement is only reached if total negotiated emissions are at most 60 billion tons of CO_2_ in the first two rounds. Equivalently, Global Wealth must be reduced from an initial level of £100 to a target level of £60 if the Global Target is to be met in the first two rounds. This target becomes more difficult to meet as the negotiations move forward, as outlined above. Every Country faces a similar decision-making problem. In each round of the global negotiation, all six Countries will be asked simultaneously: *“What percent of YOUR COUNTRY’s emissions/wealth do you demand to keep?”* If the required Global Target is met, then your group has reached an agreement; negotiations terminate and each Country receives its demand from that round. If agreement is not reached, the negotiation continues to the next round. If an agreement is not reached by the end of the 8^th^ Round of negotiations, dangerous climate change becomes unavoidable and economic costs for all Countries ensue. Each Country will then receive **10% of its initial wealth** (£3 for Rich Countries, £1 for Poor Countries).

*Example 1*. *Imagine that you are part of a negotiation group that makes decisions as follows.* In **Round 1** (Global Target=60%), all Countries demand to keep 90% of their emissions/wealth. If the Global Target were to be met, Rich Countries would receive £27 in payout and Poor Countries would receive £9 in payout. See **Screenshot 1** below, for the screen that will be seen by Poor Country 1. However, the Global Target is NOT met and negotiations continue to Round 2. In **Round 2** (Global Target=60%), demands are as follows:

- Rich Country 1 and Poor Country 1 each demand to keep 50%. If the Global Target were to be met, Rich Country 1 would receive 50% of its initial wealth (£15) and Poor Country 1 would receive 50% of its initial wealth (£5).
- Rich Country 2 and all remaining Poor Countries (2,3,4) each demand to keep 80%. If the Global Target were to be met, Rich Country 2 would receive 80% of its initial wealth (£24) and Poor Countries 2, 3, and 4 would receive 80% of their initial wealth (£8 each).

See **Screenshot 2** below. However, Global Demand = 68% > Global Target = 60%, so the Global Target is not met and negotiations continue. *Now imagine that the negotiation group continues to demand to keep emissions/wealth above the target level until the 7^th^ Round, when the relevant Global Target is 30% of emissions/wealth.* In **Round 7**, demands are as follows:

- Rich Country 1 and Poor Country 4 demand to keep 32% each.
- Rich Country 2 and Poor Countries 1, 2, and 3 demand to keep 20% each.

See **Screenshot 3**.

Hence, Global Demand = 25% ≤ Global Target = 30%. The Global Target is met. Rich Country 1 receives 32% of its initial wealth (£9.60), Rich Country 2 receives 20% of its initial wealth (£6), Poor Countries 1, 2, and 3 each receive 20% of their initial wealth (£2 each), and Poor Country 4 receives 32% of its initial wealth (£3.20). Please take a brief moment to review and understand the rules, then continue to the next page to test your understanding.

***Control questions.* Test your understanding:** *For the questions below, please check the box of the correct answer or fill in your answer on the line provided. For convenience, we summarised the main rules below:*

| **Global Target** |
| --- |
| Rounds 1-2: **60%** |
| Rounds 3-4: **50%** |
| Rounds 5-6: **40%** |
| Rounds 7-8: **30%** |

| **Country Initial Wealth** |
| --- |
| Rich Country 1, Rich Country 2: **£30** |
| Poor Country 1, Poor Country 2,  Poor Country 3, Poor Country 4: **£10** |

**1. In Round 4’s global negotiation, all members of your negotiation group demand to keep 60% of their initial emissions/wealth. What happens next?**

- *We’ve met our Global Target; each of us receives 60% of our initial wealth.*
- *Our Global Target has not been met; we continue to Round 5.*

**2. In Round 3’s global negotiation, all Rich Countries demand to keep 50% of their original emissions/wealth. If two Poor Countries demand to keep 40% and the other two Poor Countries demand to keep 60%, is agreement reached?**

- Yes
- No

*If yes, how much does each Country receive (without show-up fee)? If no, please leave blank.*

Rich Countries: £__________each

Poor Countries that demanded 60%: £__________each

Poor Countries that demanded 40%: £________each

**3. In the final Round’s global negotiation (i.e. Round 8), one Rich Country demands to keep 20% of its initial emissions/wealth and the other Rich Country demands to keep 30%. If two Poor Countries demand to keep 30% each and the other two Poor Countries demand to keep 75% each, is agreement reached?**

- Yes
- No

*How much does each Country receive as their final payout (without show-up fee)?*

Rich Country that demanded 20%: £__________

Rich Country that demanded 30%: £__________

Poor Countries that demanded 30%: £_________each

Poor Countries that demanded 75%: £__________each

*Please raise your hand when you have answered all questions, and we will come to check your answers.*

***Side Deals****.* Recall that the Global Target changes every two rounds. Before global negotiations on a new target begin, both groups of Countries (the 4 Poor and the 2 Rich) will simultaneously enter into separate Side Deals, as follows.

1. **Side Deal for Poor Countries:**

Prior to the global negotiations in Rounds 1, 3, 5, and 7, each Poor Country will enter its preferred ‘Maximum Demand’, i.e. the desired maximum percentage of emissions/wealth that each *Poor Country* may demand to keep in the two upcoming global negotiations.

The average of these four Maximum Demands will determine the ‘Agreed Maximum Demand for Poor’, which cannot be exceeded by each Poor Country in the two upcoming global negotiations.

1. **Side Deal for Rich Countries:**

At the same time, and prior to the global negotiations in Rounds 1, 3, 5, and 7, each Rich Country will enter its preferred ‘Maximum Demand’, i.e. the desired maximum percentage of emissions/wealth that each *Rich Country* may demand to keep in the two upcoming global negotiations.

The average of these two Maximum Demands will determine the ‘Agreed Maximum Demand for Rich’, which cannot be exceeded by each Rich Country in the two upcoming global negotiations.

Should a global agreement *not* be reached within the first two rounds, a new target will apply to Round 3 (Global Target=50%) and a new Agreed Maximum Demand will be determined by both Poor and Rich Countries for the two upcoming rounds (Rounds 3 and 4). This process will continue until Round 8 so long as a global agreement is not reached. Please refer to the timeline in **Screenshot 4** for a recap on the various stages of the game.

*Example 2. Imagine that you are Poor Country 1 and that you have entered into a Side Deal with the other Poor Countries. In the experiment you will see the following screen* (**Screenshot 5**).

The choices from the Side Deal for Poor Countries are shown at the top of **Screenshot 6**, which we have highlighted with a box:

- Poor Country 1 (you) chooses Maximum Demand = 100%
- Poor Country 2 chooses Maximum Demand = 66%
- Poor Country 3 chooses Maximum Demand = 33%
- Poor Country 4 chooses Maximum Demand = 0%

The resulting agreed Side Deal is that each Poor Country cannot exceed 50% demand in the two upcoming global negotiations, i.e. the Agreed Maximum Demand = 50%. (Note that the outcomes of the Side Deal for Rich Countries, which took place at the same time, are also shown in **Screenshot 6**. All Countries see these outcomes.)

*Example 3. Imagine that you are Rich Country 1 and that you have entered into a Side Deal with Rich Country 2. In the experiment you will see the following screen* (**Screenshot 7**).

The choices from the Side Deal for Rich Countries are shown at the bottom of **Screenshot 8**, which we have highlighted with a box:

- Rich Country 1 (you) chooses Maximum Demand = 75%
- Rich Country 2 chooses Maximum Demand = 25%

The resulting agreed Side Deal is that each Rich Country cannot exceed 50% demand in the two upcoming global negotiations, i.e. the Agreed Maximum Demand = 50%.

**(e) Questionnaire**

| **Question** | **Response** |
| --- | --- |
| Was the experiment difficult to understand? | Not at all difficult  Somewhat difficult  Difficult  Very difficult  Extremely difficult |
| Please select the MOST important reason for your decisions during the experiment. *Note: the questionnaire also asked for the second and third most important reasons.* | Monetary self-interest  Fairness consideration  Maximise group performance (i.e. efficiency)  Minimise time spent negotiating  Beliefs about actual (international/climate) negotiations  Past behaviour of group members  Other |
| If you could redo the experiment, how would you change your choices (if at all)? | Open-ended |
| In the scenario where 'Rich Country 1' and 'Rich Country 2' are each endowed with 30 billion metric tons in CO2 emissions (or £30) and the four Poor Countres are each endowed with 10 billion tons in CO2 emissions (or £10) each, what do you think would have been a fair initial demand (%) for each of the Rich Countries? | Open-ended (number) |
| What do you think would have been a fair initial demand (%) for each of the Poor Countries? | Open-ended (number) |
| Imagine you are in the final round of negotiation. All of the other countries in your group have made their demands and your demand could be pivotal (i.e. 'tip the scale' in terms of whether an agreement is reached or not). In this situation, what is the minimum demand (%) you would accept if you knew that your decision would change the group outcome from non-agreement to agreement? | Open-ended (number) |
| Now you will select from among five different gambles the one gamble you would like to play. The five different gambles are listed below. You must select one and only one of these gambles.  Each gamble has two possible outcomes (Event A or Event B), each with a 50% probability of occurring. Your compensation for this part of the study will be determined by: 1) which of the five gambles you select; and 2) which of the two possible events occur.  Please note that if you should select either gamble 4 or gamble 5 and Event B occurs, your losses will be deducted from your show-up fee.  For example: If you select gamble 4 and Event A occurs, you will be paid $£3.40. If Event B occurs, you will have £0.20 deducted from your £5 show-up fee.  For every gamble, each event has a 50% chance of occurring.  At the end of the study, a volunteer will be asked to flip a coin to determine whether Event A (heads) or Event B (tails) will pay out.  Please select your preferred gamble and then WRITE THE NUMBER OF THE GAMBLE YOU SELECTED ON YOUR PAYMENT SLIP. | Gamble 1: £1.00 vs. £1.00  Gamble 2: £1.80 vs. ££0.60  Gamble 3: £2.60 vs £0.20  Gamble 4: £3.40 vs. -£0.20  Gamble 5: £4.20 vs. -£0.60 |
| Are you generally a person who is fully prepared to take risks (risk prone) or do you try to avoid taking risks (risk averse)? Please select from the following options, where 0 means EXTREMELY RISK AVERSE and 10 means EXTREMELY RISK PRONE. | 0 1 2 3 4 5 6 7 8 9 10 |
| Have you ever donated money or goods to a charitable organisation? If yes, how frequently? | Very often  Often  Sometimes  Rarely  Never |
| Is global climate change a serious problem? | Extremely serious  Very serious  Serious  Somewhat serious  Not at all serious |
| Which of the following guiding principles describes your understanding of fairness best in the context of international climate negotiations? | 1. Countries with high emissions in the past should reduce more emissions. 2. Countries with high economic performance should reduce more emissions. 3. Countries should reduce their emissions in such a way that emissions per capita are the same for all countries. 4. Countries should reduce their emissions in such a way that the emissions percentage is the same for all countries. |
| How often do you recycle? | Very Often  Often  Sometimes  Rarely  Never |
| Generally speaking, would you say that most people can be trusted or that you need to be very careful in dealing with people? Please tick a box on the scale, where the value 1 means "need to be very careful" and the value 10 means "most people can be trusted". You can use the values in between to make your estimate. | 0 1 2 3 4 5 6 7 8 9 10 |
| Finally, how good are you at working with fractions (e.g. “one fifth of something”) or percentages (“e.g. “20% of something”)? | Extremely good  Very good  Good  Somewhat good  Not good at all |

**(f) Additional Empirical Analysis**

**Velocity, Dynamics, and Distributions.** In terms of agreement velocity, the most successful treatment group is the one allowing for Side Deals among the Poor (PSD), where on average the groups coordinated on the threshold shortly after the second round (Supplementary Table 1). By contrast, RSD is the treatment where agreement is most delayed (3.5 rounds on average). While ASYM and ASD are comparable in terms of the average agreement round, we note that there are two ASYM groups that failed to reach agreement altogether, consistent with the higher variance in agreement round for ASYM than for ASD. Similarly, while SYM and PSD are comparable along the former dimension, one PSD group was not successful in coordinating on the threshold, consistent with the higher variance in outcomes for PSD than for SYM.

As discussed in the manuscript, all symmetrically endowed (SYM) groups maintained at least 50% of the initial pie, which is remarkably efficient given that the maximum attainable proportion of global wealth is 60%. However, the PSD treatment is the most successful in securing agreement under maximally efficient conditions (i.e. in Rounds 1-2, before the target shrinks), though we do not have the power to detect a statistically significant difference between groups’ success rates within the first two periods. Regardless, in accordance with (1), endogenous demand restrictions (i.e. binding Side Deals) on a larger number of low-emission “poor” players appear to be more successful in inducing coordination than similar restrictions on a smaller number of high-emission “rich” players if we are concerned with maximizing the global pie. Importantly, unlike (1), we do not find conclusive evidence that outcomes in treatments containing Side Deals improve upon global negotiations that occur among asymmetric actors in the absence of Side Deals, in terms of either agreement velocity or demands (at both the individual and the group levels).

Supplementary Figures 1 and 2 provide visual representations of the above statistics in addition to the demand dynamics across treatments. The early disparity in agreement rate across treatments is clear, as is the tendency of average group demands to respond to the declining values of the Global Target *T* (from 60% to 30%) by clustering, although with some variance, around these values.

Across successful asymmetric groups, the average difference between Rich and Poor demands in the successful round of negotiation is 10.65 percentage points (p<0.01). This average demand distribution translates to a final average income of £15.63 for Rich players and £6.28 for Poor players, and a final wealth distribution of 27.7% of global wealth for Rich Countries and 11.1% of global wealth for Poor Countries. Note that this subtle shift in the wealth distribution occurs solely in the negotiation over emissions reductions (i.e. it is independent of international wealth and technology transfers pervasive in climate change negotiations).

Moreover, in PSD and RSD, the standard error among players in the group who formed the Side Deal (2.45 in PSD, and 3.86 in RSD) is smaller than it is for the subgroup without constraints (6.27 in PSD, and 4.28 in RSD; Supplementary Figure 3). Therefore, in the case of the side agreements among the US, the EU, and China, we would expect low-emission countries to experience a wider variance in proposed emissions limits.

**Questionnaire Analysis.** Immediately following the experiment, subjects were asked a series of questions to gather demographic information, preferences (i.e. for fairness, risk, environment), and motivations in the experiment.

We look at players’ primary decision-making motivations, acknowledging that the *ex post* nature of the questionnaire may create dependence of answers on dynamics and outcomes of the game played previously. When asked what is the most important motivation behind their decisions in the game, most claimed to have been primarily motivated by group efficiency (36.3% of subjects) or money (29.1% of subjects), with fairness (19.6% of subjects) following close behind. The rest were motivated by time minimization (7.5% of subjects), beliefs about actual climate negotiations (3.6% of subjects), and the past behavior of group members (3.6% of subjects). If money was a subjects’ primary motivation, she initially demanded 6.9 percentage points more than if her primary motivation were not money (p=0.001).

We do not find that stating fairness as one’s primary motivation influences one’s initial demand in the SYM treatment. However, when we introduce asymmetric endowments, fairness influences demands considerably. Poor players who stated fairness as their primary motivation (22% of Poor) demanded about 4.5 percentage points more in Round 1 than those who stated another motivation (70.1 percent vs. 65.8 percent, p=0.094), consistent with the self-serving notion of fairness found in ultimatum games with asymmetric payoffs (10). Likewise, Rich players who stated fairness as their primary motivation (16% of Rich) demanded almost 10 percentage points less than those with other motivations (61.7 percent vs. 52.0 percent, p=0.025), consistent with social welfare preferences (11).

Additionally, we asked subjects what is the minimum demand they would accept if they were a pivotal player in the final round of negotiation, i.e. when the Global Target is 30%. The average minimum acceptable demand is 30.2 percent (SD=16.8) of one’s endowment, and this is not largely dependent on whether one was a Poor (mean=31.9, SD=17.2) or Rich (mean=28.5, SD=17.1) player.

We also ask a series of questions to elicit our subjects’ risk and social preferences. Using a variant of the incentive compatible risk preference elicitation question posed in (7, 8)—where 1 represents a certain outcome (50% chance of £1 vs. 50% chance of £1) and 5 represents the most risky outcome (50% chance of £4.20 and 50% chance of -£0.60)—subjects’ average selection is 3.77 (SD=1.33). When asked to self-assess the extent to which they are risk prone on a scale from 0 to 10 (where 10 is extremely risk prone; see 12), subjects’ average selection is 5.38 (SD=2.11). To assess subjects’ altruism, we ask them to state the frequency with which they donate to charity: 6.9% of subjects give to charity *very often*, 17.7% give to charity *often*, 45.8% give to charity *sometimes*, 28.1% give to charity *rarely*, and 1.6% *never* give. We also asked subjects the extent to which they think others can be trusted on a scale from 1 (low trust) to 10 (high trust), and the mean response is 4.9 (SD=2.1). To get a reading of subjects’ preferences for the environment, we asked how often the subjects recycle. In our pool, 27.4% claim to recycle *very often*, 39.0% recycle *often*, 19.1% recycle *sometimes*, 4.8% recycle *rarely*, and 9.8% *never* recycle. Additionally, when asked their opinion on the severity of the problem of climate change, 35.0% of subjects responded that it is *extremely serious*, 36.0% that it is *very serious*, 19.6% that it is *serious*, 8.2% that it is *somewhat serious*, and 1.3% that it is *not at all serious*. Group-level heterogeneity in self-reported charitable spending and ‘green’ preferences do not play a significant role in determining subjects’ decision-making nor the velocity of agreement in the game, contrary to the assertion that heterogeneity of preferences increases the transaction costs associated with (and therefore decreases the likelihood of) reaching agreement (13).

To gauge whether subjects understood the experiment, we ask the extent to which the instructions are comprehensible and ask for an evaluation of subjects’ own ability to work with fractions. Subjects appear to have understood the experiment, with only six subjects (i.e. less than two percent) stating that the experiment was (very) difficult to understand. Similarly, only 2.3% of subjects claim they are *not at all good* with fractions, while 9.2% are *somewhat* *good* with fractions, 27.8% are *good* with fractions, 37.3% are *very good* with fractions, and 23.5% are *extremely good* with fractions.

***Risk Preferences.*** To further understand the dynamics underpinning group coordination, we investigate the role of individual risk preferences in predicting behavior in the negotiation. As expected, we find that risk aversion reduces demand, on average. In Supplementary Table 2, we display the effects of risk preferences on individual demand in a panel OLS regression. *Risk* is measured on a scale from 1 to 5, where 1 represents the most risk-averse gamble option—a gamble with payout certainty—and 5 represents the most risk-seeking option.

Supplementary Table 2 demonstrates that the effect of the risk parameter on demand is robust with respect to inclusion of various controls. The initial inclusion of controls—including demographics, stated motivation, Global Target, and treatment group assignment—reduces the magnitude of the effect from 1.68 to 1.24 percentage points per one-point increase on the risk scale. However, the magnitude of the effect is restored when we additionally account for the role of threshold (even) rounds—or rounds in which a failure to reach agreement results in negative group-level consequences— which have a large negative effect on demand, as expected.

We further investigate the role of threshold effects through the interaction term between threshold rounds and risk preferences. Since threshold rounds threaten to diminish global welfare, we expect risk-averse individuals to err on the side of caution by demanding less than risk-prone individuals in these rounds. In regression four of Supplementary Table 2, we see that the state of being in a threshold round reduces individual demand by almost six percentage points on average. However, the positive coefficient for the interaction term—which is significant both here (p=0.068) and when using self-reported risk preferences as the independent variable (p=0.036; see Supplementary Table 3)—indicates that this threshold effect is less strongly negative the more risk seeking is the individual.

While risk preferences are important predictors in the game, the question remains as to the interpretation of such results when considering actions taken by countries. We suffice to say here that risk preferences may potentially be an important and understudied predictor of (climate) bargaining strategies, whether they are risk preferences of the negotiators themselves or broader cultural parameters. For example, countries may signal risk attitudes through policies or military and geopolitical strategies, in turn providing information on their bargaining strategies. Our results indicate that risk preferences in bargaining may be a promising avenue for future research.

***Self-Serving Bias.*** Our data allows for empirical estimation of self-serving bias (14, 15). In the questionnaire described above, subjects were asked a series of survey questions, one of which pertained to their perspectives on equity in the context of climate change. To test for self-serving bias, we look at the average marginal effects of logit regressions where the dependent variables are dummies for whether the particular equity perspective in question has been selected, and the independent variables are indicators for subjects’ nationality (United States, European Union, or China). Controlling for whether subjects played the role of a Rich or Poor Country in the experiment, we find that European subjects were less likely to identify with the perspective that “Countries with high emissions in the past should reduce more emissions” by 12.95% (p=0.038), although they were somewhat more likely than non-Europeans to identify with the perspective that “Countries with high economic performance should reduce more emissions” by 9.7% (p=.123). Additionally, we find that Chinese subjects were less likely to select “Countries with high economic performance should reduce more emissions” by 15.64% (p=0.055). We do not find definitive evidence of self-serving bias among Americans in our sample; however, American subjects were less likely to identify with the perspective that “Countries should reduce their emissions in such a way that emissions per capita are the same for all countries” than non-Americans by 13.8%, though the effect is not quite significant at conventional levels (p=0.140).

**Supporting Analysis: Robustness.** To account for the maximum demand imposed in the experimental design, we run a panel Tobit regression (see Supplementary Tables 4 and 5) to complement the panel OLS regressions previously discussed (see Table 3 in the manuscript and Supplementary Table 2). These regressions place an upper limit of 100 on individual demands. Since subjects may wish to demand more than 100 percent of their endowed share of global emissions, the Tobit regressions censor the dependent variable from above at 100. Note that it is not necessary to censor the dependent variable from below since none of the experimental subjects demanded zero emissions in the game. The results of the Tobit regressions align closely with those of the OLS regressions, providing a simple robustness check of the conditional demand result and the influence of risk preferences on individual demands.

We run an additional panel OLS regression (see Supplementary Table 3), replacing the incentive compatible risk preference with a stated preference for risk as our dependent variable. Again, the results are qualitatively similar to those in Supplementary Table 2. While the incentive compatible risk responses map preferences on a scale from 1 to 5, the stated risk responses map preferences on a scale from 0 to 10. Standard errors are slightly inflated relative to the OLS regression on the incentive compatible risk preference. However, the results for Regressions 1-3 across the two tables are qualitatively similar. Interestingly, the results for Regression 4 indicate a positive though non-significant effect of risk preference on demand in the game, while the interaction between threshold round and risk becomes significant. That is, subjects who indicate a higher risk tolerance demand more in threshold rounds than do those who report lower risk tolerance.

**References**

1. Smead R., Sandler R., Forber P., Basl J. A bargaining game analysis of international climate negotiations. *Nat. Clim. Chang.* **4**, 442-445 (2014).
2. Friedlingstein, P. *et al.* Persistent growth of CO_2_ emissions and implications for reaching climate targets. *Nat. Geosci.* **7**, 709-715 (2014).
3. Tavoni, A., Dannenberg, A., Kallis, G., & Loschel, A. Inequality, communication, and the avoidance of disastrous climate change in a public goods game. *Proc. Natl Acad. Sci. USA* **108**, 11825-11829 (2011).
4. Milinski, M., Sommerfeld, R., Krambeck, H., Reed, F. & Marotzke J. The collective-risk social dilemma and the prevention of simulated dangerous climate change. *Proc. Natl Acad. Sci. USA*, **105,** 2291-2294 (2008).
5. Burton-Chellew, M., May, R., West, S. Combined inequality in wealth and risk leads to disaster in the climate change game. *Clim. Chang.***120**, 815-830 (2013).
6. Gampfer, R. Do individuals care about fairness in burden sharing for climate change mitigation? Evidence from a lab experiment. *Clim. Chang.* **124**, 65-77 (2014).
7. Eckel, C. & Grossman, P. Forecasting risk attitudes: an experimental study using actual and forecast gamble choices. *J. Econ. Behav. Organ.* **68,** 1-17 (2008).
8. Binswanger, H. Attitudes toward risk: experimental measurement in rural India. *Am. J. Agr. Econ.* **62**, 395-407 (1980).
9. Fischbacher, U. z-Tree: Zurich toolbox for ready-made economic experiments. *Exp. Econ.* **10,** 171-178 (2007).
10. Kagel, J., Kim, C, & Moser, D. Fairness in ultimatum games with asymmetric information and asymmetric payoffs. *Game. Econ. Behav.* **13**, 100-110 (1996).
11. Charness, G. & Rabin, M. Understanding social preferences with simple tests. *Q. J. Econ.* **117**, 817-869 (2002).
12. Dohmen, T., Falk, A., Huffman, D., Sunde, U., Schupp, J., & Wagner, G. G. Individual risk attitudes: Measurement, determinants, and behavioural consequences. *J. Eur. Econ. Assoc.* **9**, 522-550 (2011).
13. Libecap, G. Addressing global environmental externalities: transaction costs considerations. *J. Econ. Lit.* **52**, 424-479 (2014).
14. Lange A. & Vogt C. Cooperation in international environmental negotiations due to a preference for equity. *J. Pub. E.* **87**, 2049-2067 (2003).
15. Lange, A., Löschel, A., Vogt, C., & Ziegler, A. On the self-interested use of equity in international climate negotiations. *Eur. Econ. Rev.* **54**, 359-375 (2010).

**Screenshots from game interface (from the experimental instructions)**


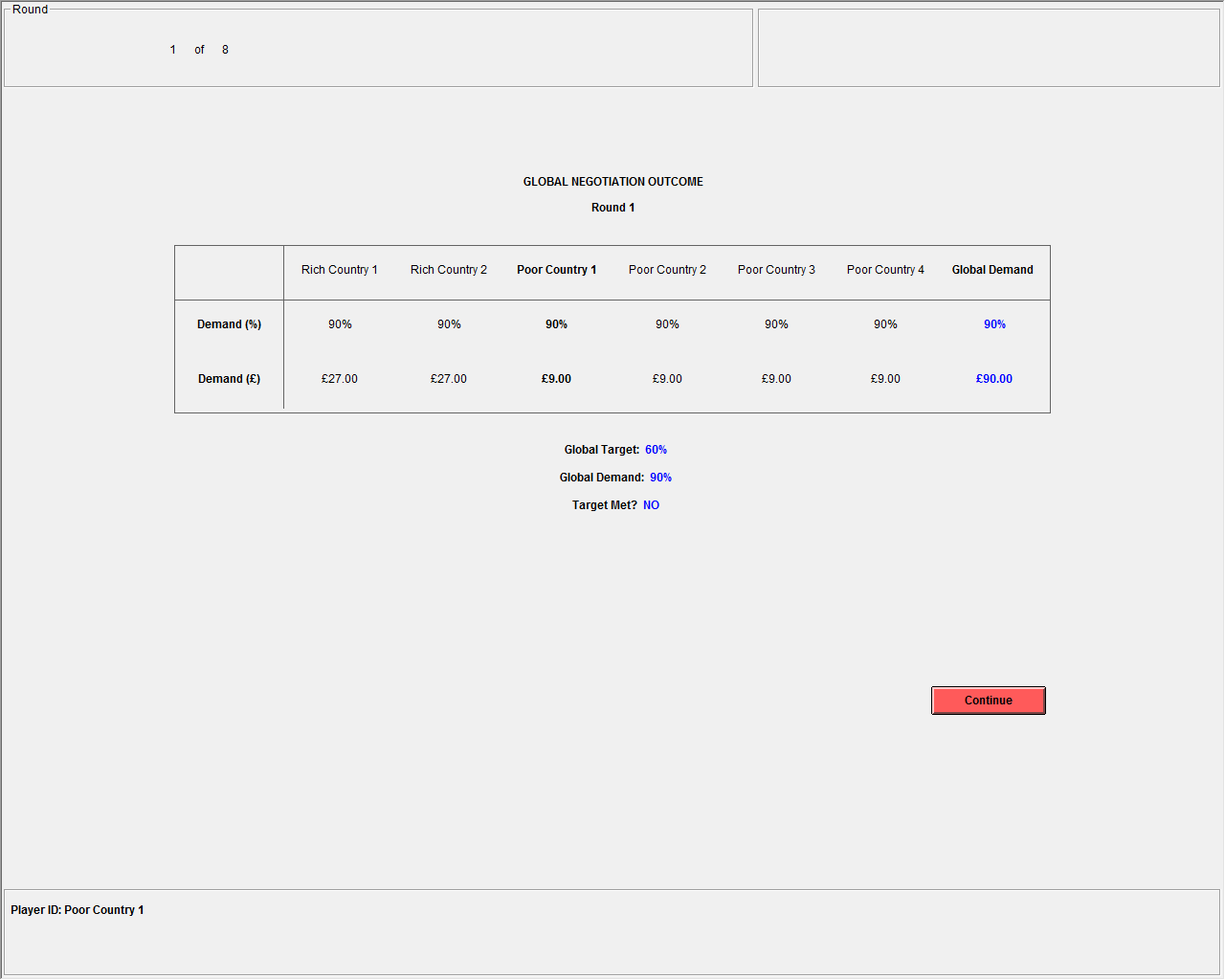


**Screenshot 1 |** Outcome screen presented to Poor Country 1 in Round 1 of the Global Negotiation if all group members demand to keep 90% of their initial wealth/emissions in ASYM, PSD, RSD, and ASD. The Global Demand exceeds the Global Target of 60% in Round 1 and negotiations continue to Round 2.


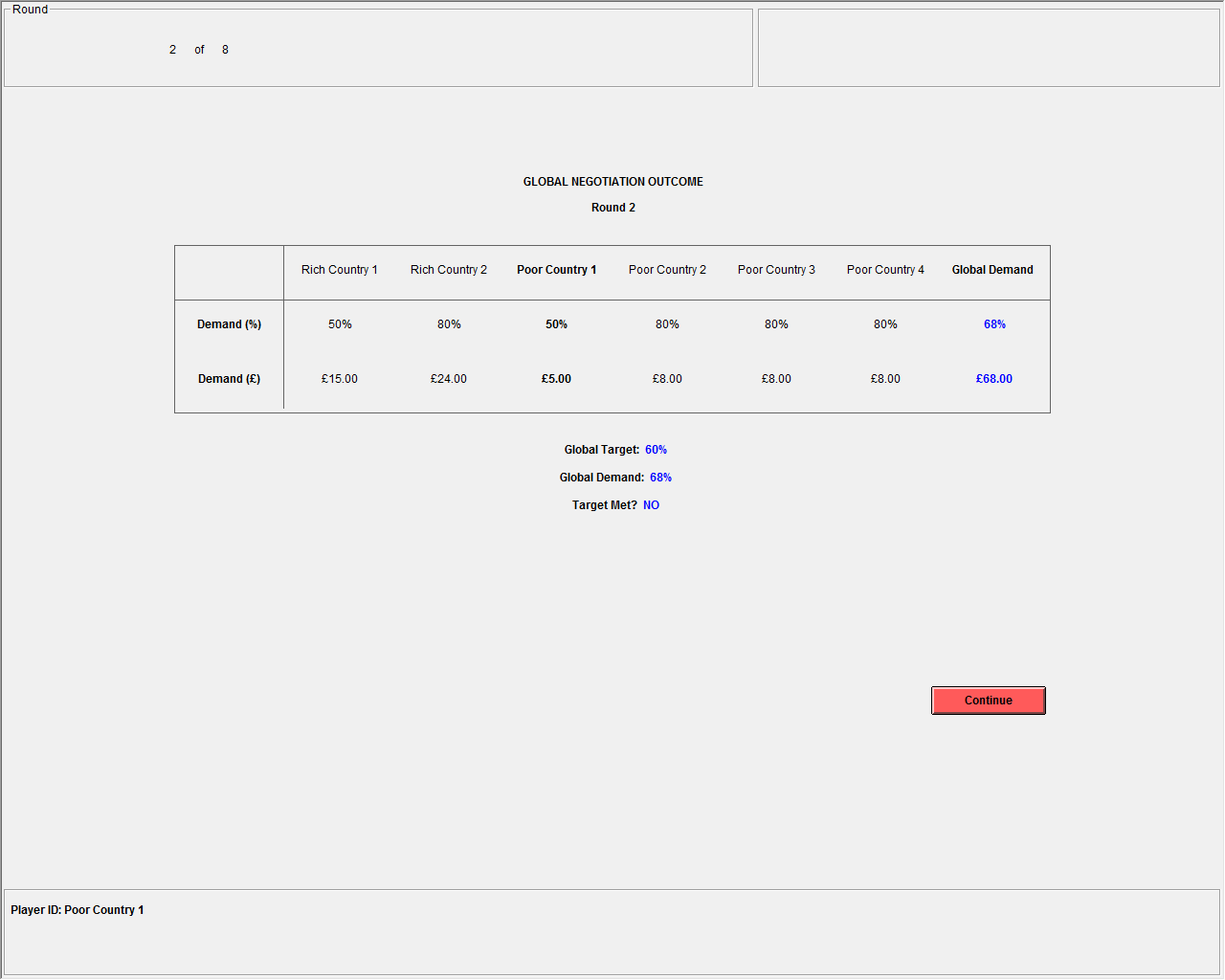


**Screenshot 2 |** Outcome screen presented to Poor Country 1 in Round 2 of the Global Negotiation if Rich Country 1 and Poor Country 1 demand to keep 50% of their initial wealth/emissions and all other players demand to keep 80% of their initial wealth/emissions. The Global Demand exceeds the Global Target of 60% in Round 1 and negotiations continue to Round 3.


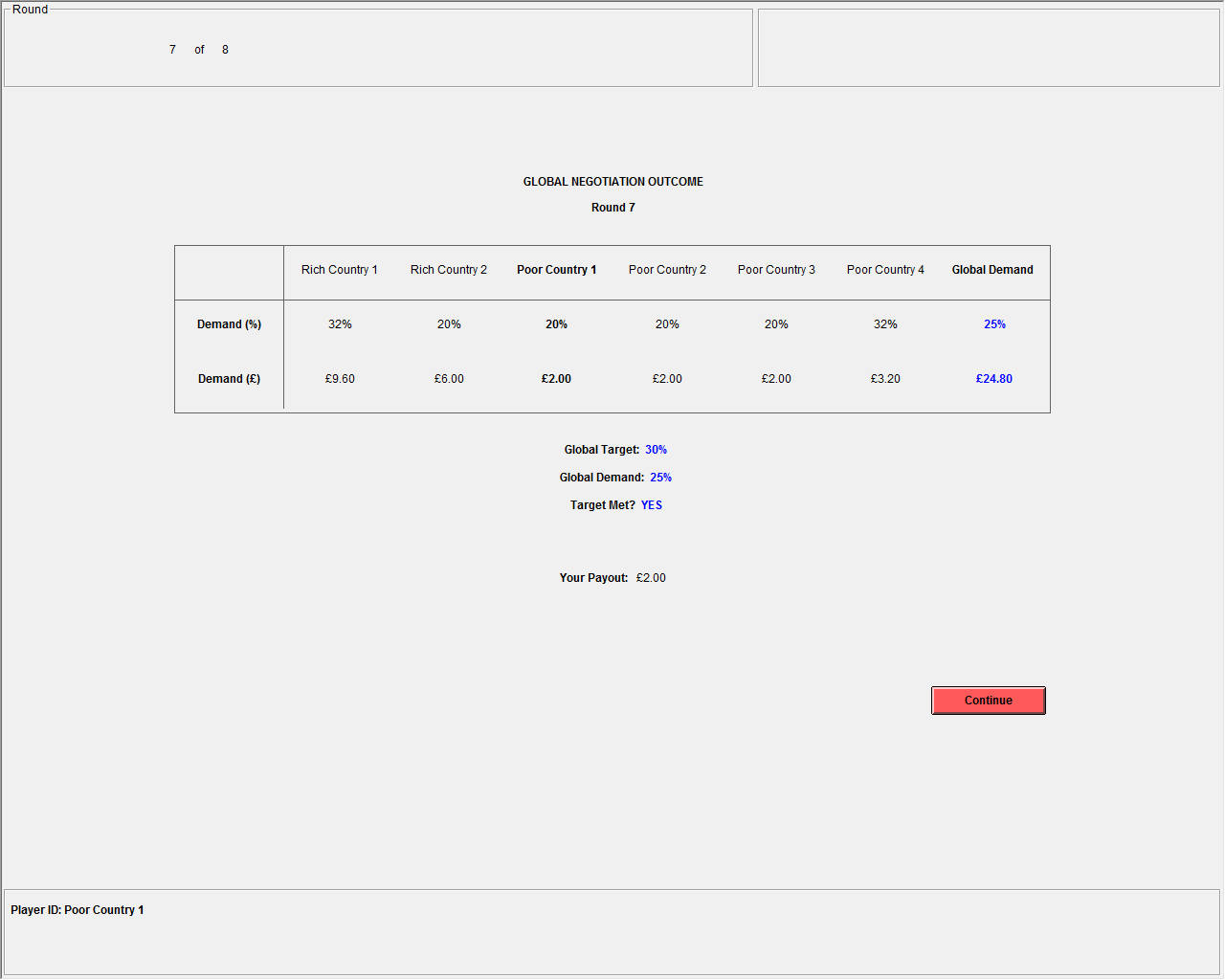


**Screenshot 3 |** Outcome screen presented to Poor Country 1 in Round 7 of the Global Negotiation if Rich Country 1 and Poor Country 4 demand to keep 32% of their initial wealth/emissions and all other players demand to keep 20% of their initial wealth/emissions. The Global Demand is less than the Global Target of 30% in Round 7. Each player receives her demand and negotiations terminate.


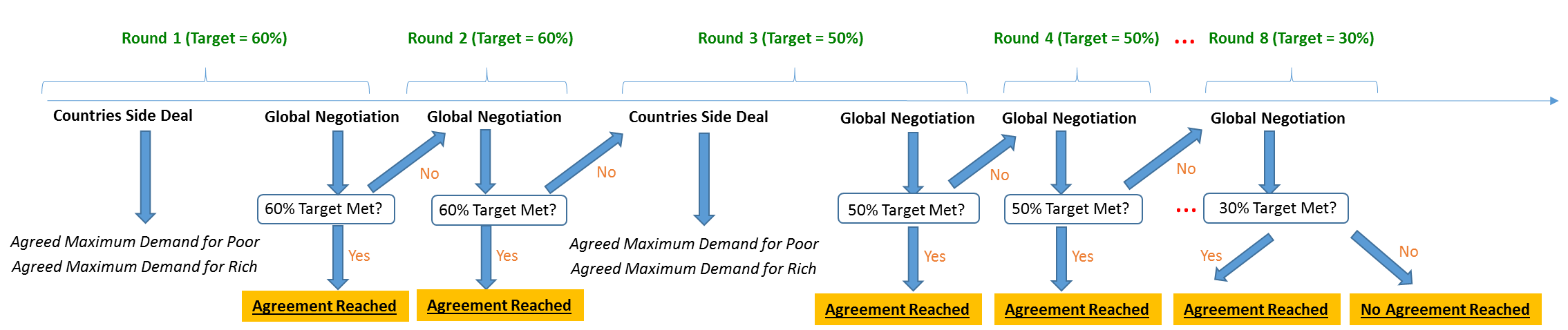


**Screenshot 4 |** A schematic representation of the stages in treatment ASD. In even-numbered rounds there is only one stage (Global Negotiation), while in odd-numbered rounds that stage follows a Side Deal stage. The same applies to PSD and RSD, except that the Side Deal in those treatments are determined by (and pertain only to) Poor and Rich Countries, respectively.


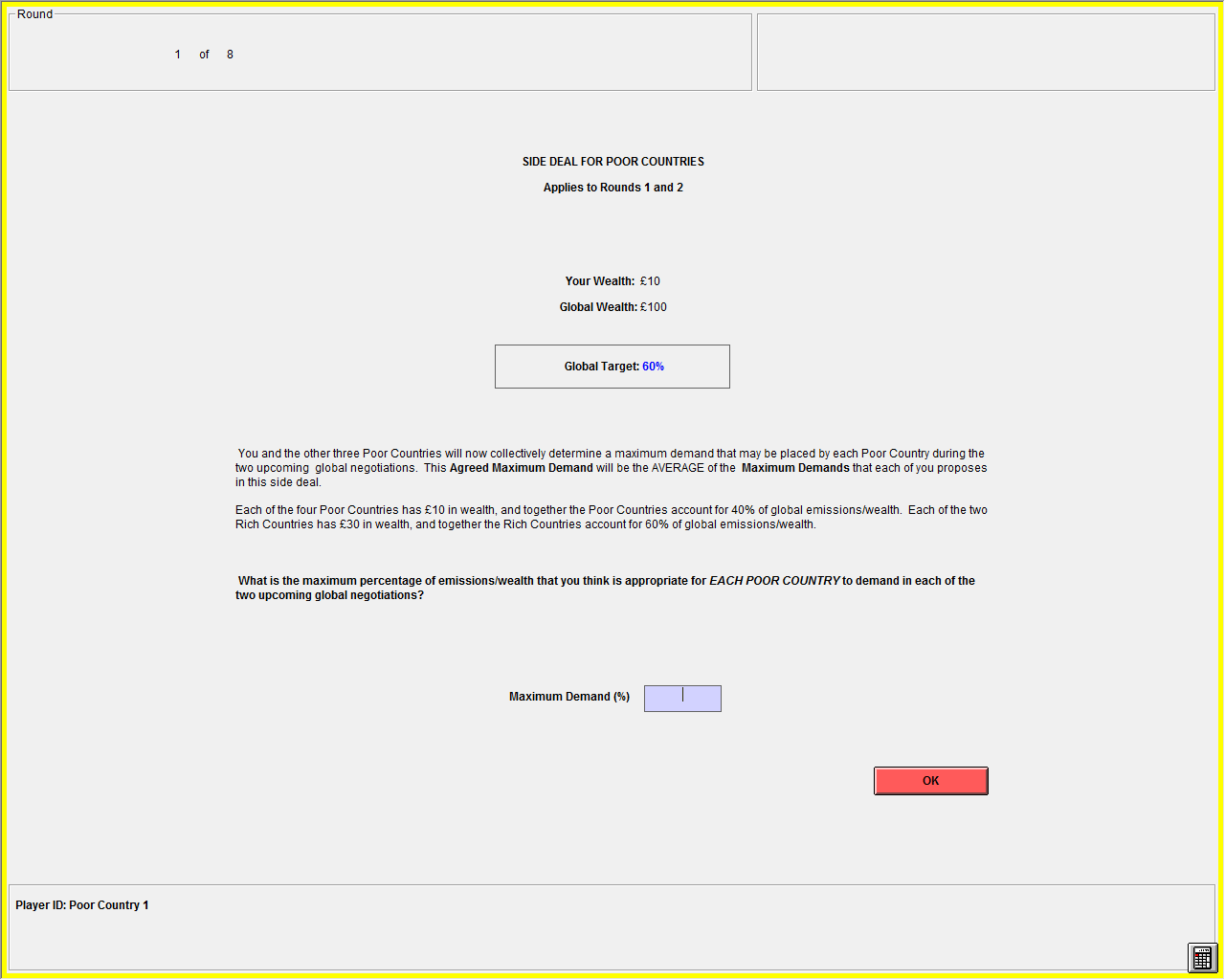


**Screenshot 5 |** Input screen presented to Poor Country 1 to designate a preferred Maximum Demand in the Poor Countries’ Side Deal prior to Rounds 1 and 2 of the Global Negotiation.


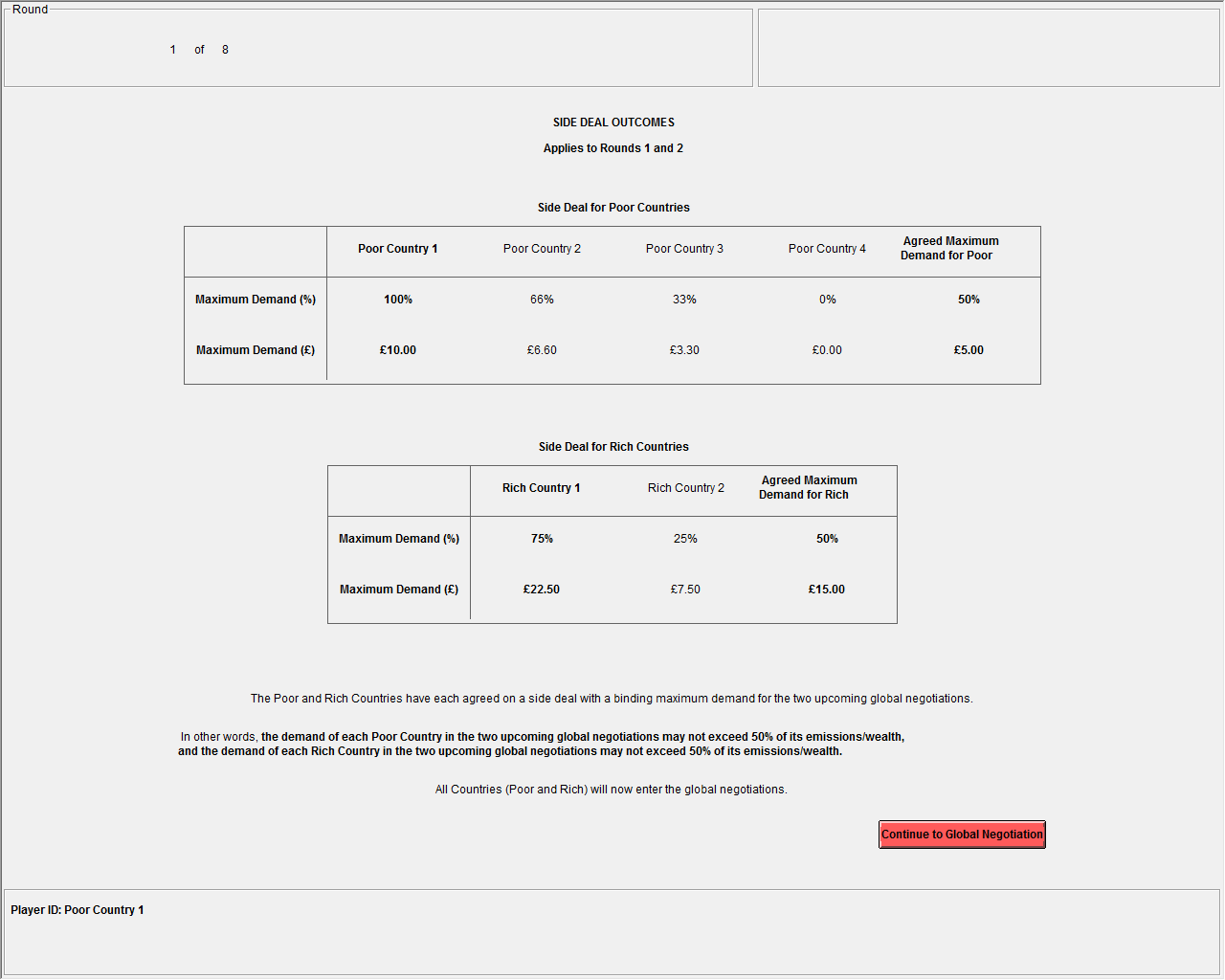


**Screenshot 6 |** Outcome screen presented to Poor Country 1 displaying the selected Maximum Demands of all other players in her group. The red box is included in the Experimental Instructions to highlight the relevant Agreed Maximum Demand from the perspective of Rich Country 1, though it does not appear on screen during the experiment. Agreed Maximum Demands for both Rich Countries and Poor Countries are revealed to all group members prior to the subsequent Global Negotiation stages.


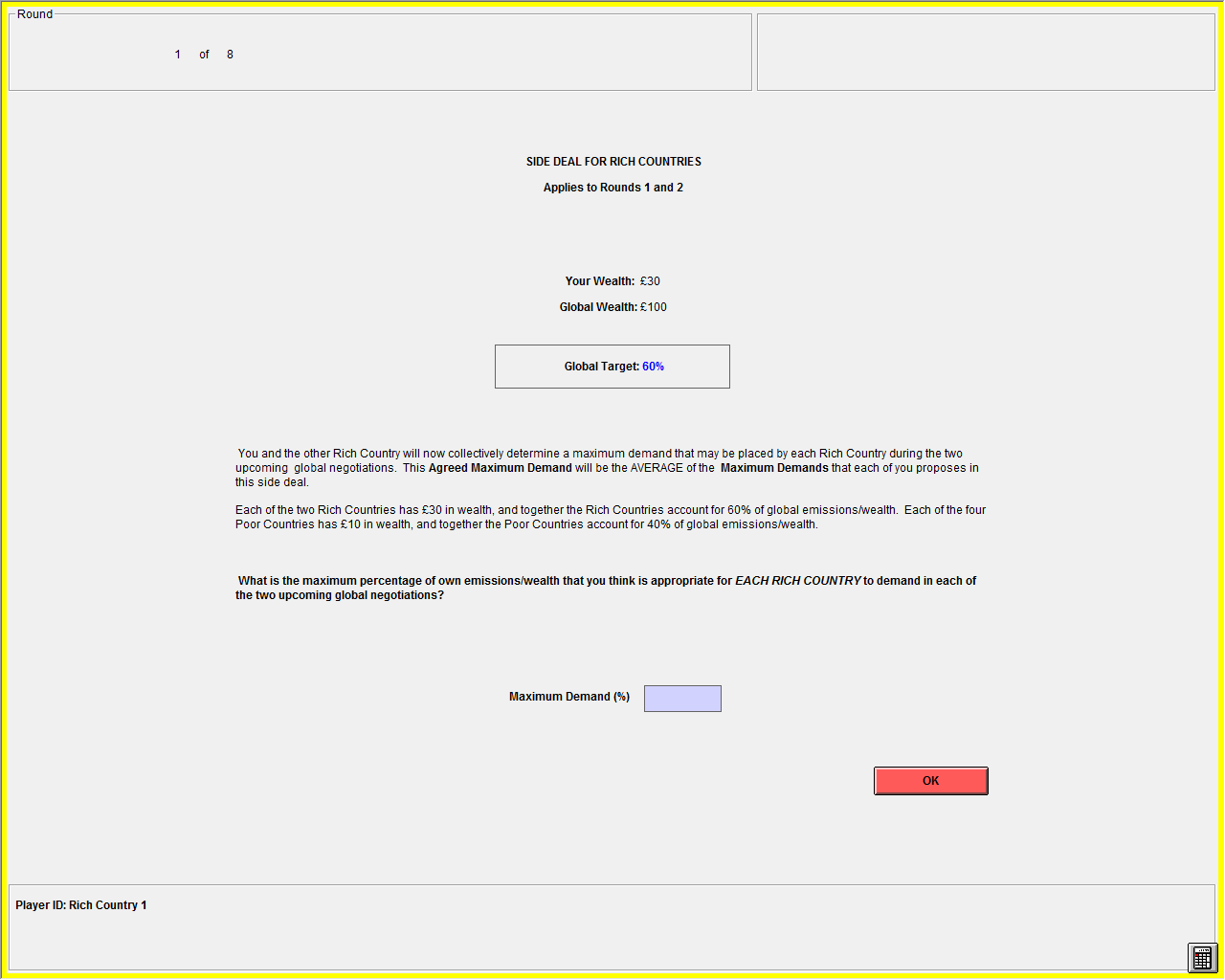


**Screenshot 7 |** Input screen presented to Rich Country 1 to designate a preferred Maximum Demand in the Rich Countries’ Side Deal prior to Rounds 1 and 2 of the Global Negotiation.


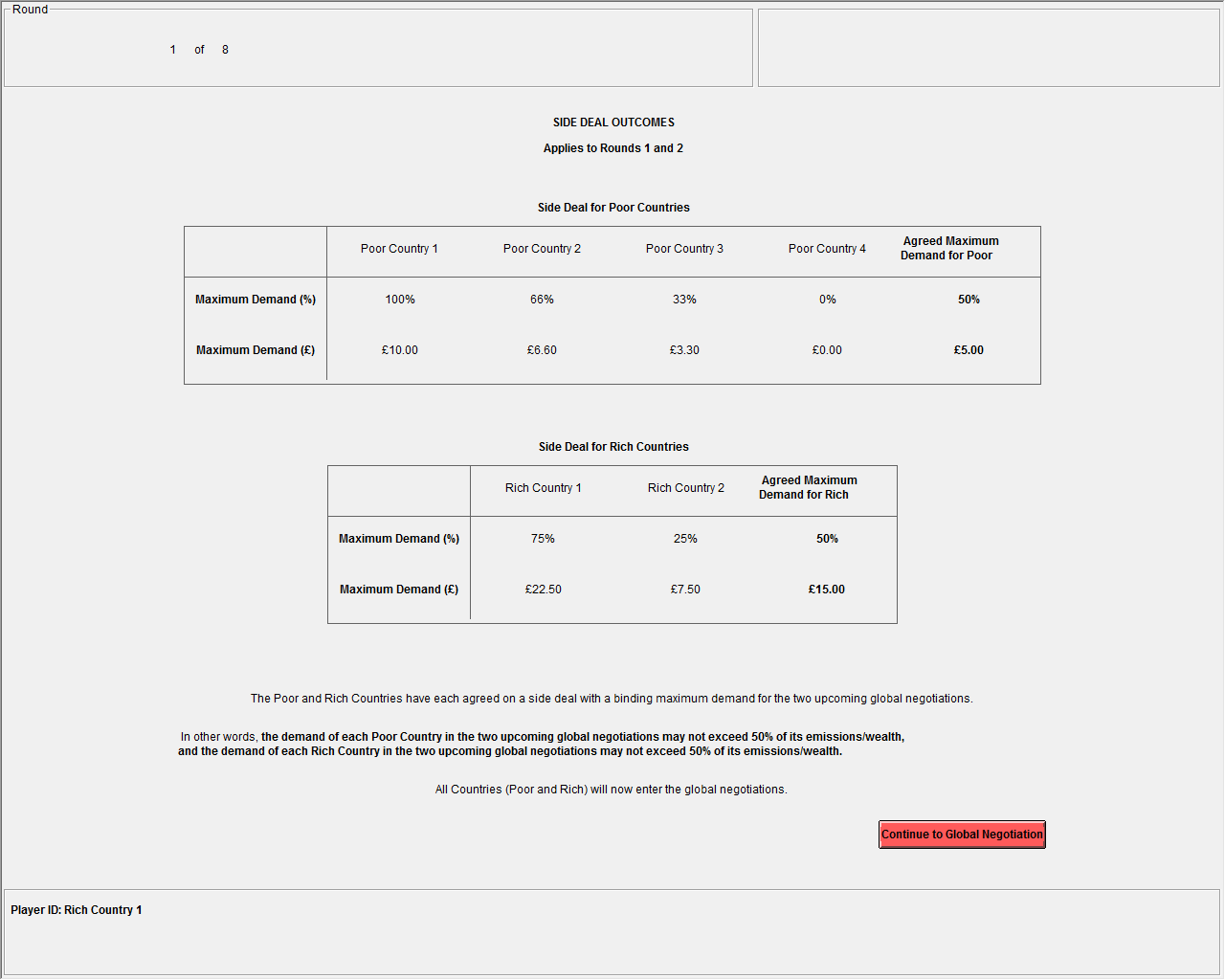


**Screenshot 8 |** Outcome screen presented to Rich Country 1 displaying the selected Maximum Demands of all other players in her group. The red box is included in the Experimental Instructions to highlight the relevant Agreed Maximum Demand from the perspective of Rich Country 1, though it does not appear on screen during the experiment. Agreed Maximum Demands for both Rich Countries and Poor Countries are revealed to all group members prior to the subsequent Global Negotiation stages.

**Supplementary Figures**


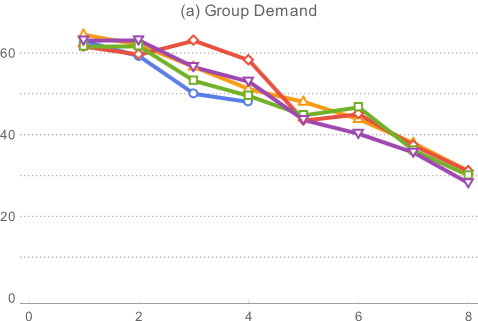

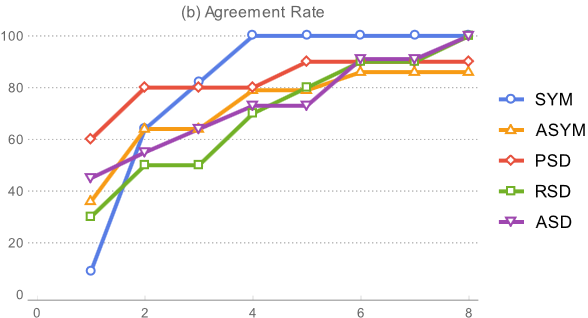

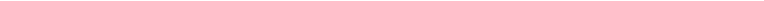


**Supplementary Figure 1 | Group demand over time (a) and agreement rate (b).** Figure 1a illustrates group demand dynamics, while Figure 1b shows the percent of groups that reached agreement, by round and treatment. Data points in 1(a) should be weighted differently according to the number of groups remaining in the negotiation. For instance, 60% of SYM groups reach agreement in Round 1, so the average group demand for SYM in Round 2 represents the average of the 40% of groups who continued to negotiate in Round 2.

1. **(b)**


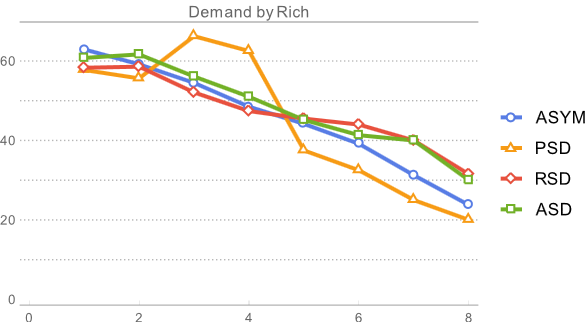

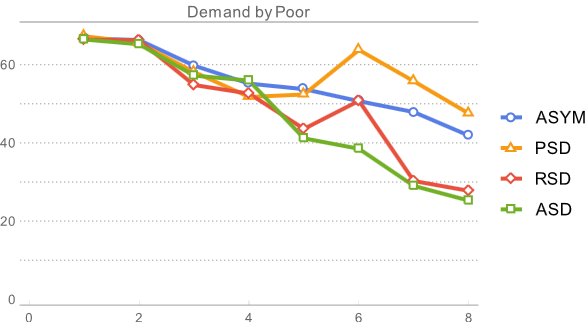


**Supplementary Figure 2 | Demands over time by treatment, for the Poor (a) and for the Rich (b).** The figure illustrates individual demands over time for both player types.

**Supplementary Figure 3 |** Average demands (and standard errors bars) by Poor (blue) and Rich (red) negotiators in agreement round of successful groups (i.e. groups who reached agreement in the first two rounds).

**Supplementary Tables**

| **Supplementary Table 1 \| Agreement velocity (average round in which negotiations terminated) and failures (number of groups that failed to reach an agreement), by treatment** | | | | | |
| --- | --- | --- | --- | --- | --- |
|  | SYM | ASYM | PSD | RSD | ASD |
| Velocity | 2.455 | 3.071 | 2.300 | 3.400 | 3.091 |
|  | (0.934) | (2.556) | (2.359) | (2.413) | (2.548) |
| Failures | 0 | 2 | 1 | 0 | 0 |
| *Groups* | 11 | 14 | 10 | 10 | 11 |

**Supplementary Table 2 | Risk preferences and individual demands**

|  | (1)  No Controls | (2)  With Controls | (3)  With Threshold Control | (4)  With Threshold Interaction |
| --- | --- | --- | --- | --- |
| Risk | 1.680*** | 1.241** | 1.769*** | 1.541** |
|  | (0.535) | (0.562) | (0.666) | (0.678) |
| Threshold Round |  |  | -3.989*** | -5.737*** |
|  |  |  | (0.550) | (1.015) |
| Threshold Round * Risk |  |  |  | 0.476* |
|  |  |  |  | (0.276) |
| Constant | 52.198*** | 58.097*** | 61.459*** | 62.294*** |
|  | (2.270) | (2.631) | (3.105) | (3.069) |
| *Groups* | 54 | 54 | 34 | 34 |
| *Subjects* | 324 | 324 | 204 | 204 |
| *Obs* | 930 | 930 | 810 | 810 |
| The table displays the results of a panel OLS regression with errors clustered at the group level, where the dependent variable is individual demand. The risk question posed to subjects is based on the incentive-compatible risk preference elicitation gambles in (7, 8). Robust standard errors are reported in parentheses. *Threshold Round* is a dummy equal to 1 if the present round is the threshold round before a decline in the Global Target (i.e. an even round). The number of observations reduces with the threshold control since 20 groups who reach agreement in Round 1 will not experience variation in the *Threshold Round* control and are thus excluded from the regression. Controls include gender, Annex 1 nationality, stated primary motivation, Global Target, and treatment group assignment. ***p<0.001, **p<0.05, *p<0.10  **Supplementary Table 3 \| Stated risk preferences and individual demands**   \|  \| (1)  No Controls \| (2)  With Controls \| (3)  With Threshold Control \| (4)  With Threshold Interaction \| \| --- \| --- \| --- \| --- \| --- \| \| Stated Risk \| 0.697** \| 0.728** \| 0.673* \| 0.433 \| \|  \| (0.313) \| (0.305) \| (0.355) \| (0.350) \| \| Threshold Round \|  \|  \| -3.987*** \| -6.703*** \| \|  \|  \|  \| (0.550) \| (1.505) \| \| TR * Stated Risk \|  \|  \|  \| 0.496** \| \|  \|  \|  \|  \| (0.236) \| \| Constant \| 54.702*** \| 54.510*** \| 64.652*** \| 65.954*** \| \|  \| (1.811) \| (2.195) \| (2.746) \| (2.667) \| \| *Groups* \| 54 \| 54 \| 34 \| 34 \| \| *Subjects* \| 324 \| 324 \| 204 \| 204 \| \| *Obs* \| 930 \| 930 \| 810 \| 810 \|   The dependent variable in this regression is individual demand. Stated Risk is measured on a scale from 0 to 10 and comes from the general risk question asked in the German Socioeconomic Panel (SOEP; see 12). Robust standard errors are reported in parentheses. Controls include gender, Annex 1 nationality, stated primary motivation, Global Target level, and treatment group assignment. ***p<0.001, **p<0.05, *p<0.10 | | | | |

**Supplementary Table 4 | Conditional demands of Poor and Rich (Tobit)**

|  | Poor Demand | Rich Demand |
| --- | --- | --- |
| Rich Cooperated | 4.074*** | 0.766 |
|  | (1.301) | (2.024) |
| Poor Cooperated | -0.265 | 2.420*** |
|  | (0.740) | (0.805) |
| Constant | 59.397*** | 55.995*** |
|  | (4.464) | (5.315) |
| *Groups* | 26 | 26 |
| *Subjects* | 104 | 52 |
| *Obs* | 356 | 178 |
| *Controls* | Yes | Yes |
| The table displays the results of a panel Tobit regression, where the dependent variable indicates the percentage demanded of one's initial endowment. The independent variables represent the number of Rich and Poor Country representatives (respectively) who cooperated in the prior round by demanding less than or equal to the Global Target. Controls include gender, Annex 1 nationality, stated primary motivation, Global Target, and the difference between the group demand and the target in the prior round of negotiations. There are 26 groups in heterogeneous treatments that negotiated past the first period, and these are the groups considered here. Robust errors are clustered at the group level. Standard errors are reported below estimates in parentheses. ***p<0.001, **p<0.05, *p<0.10 | | |

**Supplementary Table 5 | Risk preferences and individual demands (Tobit)**

|  | (1) | (2) | (3) | (4) |
| --- | --- | --- | --- | --- |
|  | Risk | With Controls | With Threshold Interaction | With Threshold Interaction |
| Risk | 1.659*** | 1.269** | 1.805*** | 1.575** |
|  | (0.536) | (0.541) | (0.604) | (0.635) |
| Threshold Round |  |  | -4.015*** | -5.775*** |
|  |  |  | (0.563) | (1.613) |
| Threshold Round * Risk |  |  |  | 0.480 |
|  |  |  |  | (0.412) |
| Constant | 52.429*** | 58.142*** | 61.541*** | 62.382*** |
|  | (2.135) | (2.910) | (3.340) | (3.417) |
| *Groups* | 54 | 54 | 34 | 34 |
| *Subjects* | 324 | 324 | 204 | 204 |
| *Observations* | 930 | 930 | 810 | 810 |
| The table displays the results of a panel Tobit regression, where the dependent variable is individual demand. The risk question posed to subjects is based on established incentive-compatible risk preference elicitation gambles (7, 8). Robust standard errors are reported in parentheses. *Threshold Round* is a dummy equal to 1 if the present round is the threshold round before a decline in the Global Target (i.e. an even round). The number of observations reduces with the threshold control since 18 groups who reach agreement in Round 1 will not experience variation in the *Threshold Round* control and are thus excluded from the regression. Controls include gender, Annex 1 nationality, stated primary motivation, Global Target level, and treatment group assignment. ***p<0.001, **p<0.05, *p<0.10 | | | | |

1. Geography and Environment Department, London School of Economics and Political Science, Houghton Street, London, WC2A 2AE. [↑](#footnote-ref-1)
2. Grantham Research Institute, London School of Economics and Political Science, Houghton Street, London, WC2A 2AE.

   ^a^To whom correspondence should be addressed. Email: [a.tavoni@lse.ac.uk](mailto:a.tavoni@lse.ac.uk). The authors contributed equally to this work. [↑](#footnote-ref-2)
3. “Rich” and “Poor” subjects may behave differently than they would in a symmetric setup, where such labels are not assigned, as indicated in (1). Therefore, we also introduced a symmetric treatment where subjects receive equal endowments, which serves as a baseline towards which wealth heterogeneity can be assessed. We note, however, that division into two groups—regardless of labels—may be considered an additional departure of the ASYM treatment to the SYM treatment. However, an arbitrary division of SYM groups into two groups of unequal sizes may have further confused the instructions, so we opted to ignore this slight departure and assume it holds negligible bearing on the results. [↑](#footnote-ref-3)
4. In addition to mimicking real-world heterogeneity, our experiment shares the rich-poor dichotomy with the theoretical investigation in (1)—whose pertinent findings inspired our behavioral investigation—and with related experimental literature pertaining to allocation of emissions in the context of climate change (e.g., 3-6). Our experimental design encapsulates additional features from similar experiments, namely (3). In their experiment, groups composed of six (asymmetrically endowed) players aim to avoid the losses associated with catastrophic climate change in a dynamic framed experiment. However, unlike (3), we do not impose a set number of rounds, and we do not vary the probability of climate catastrophe if the target is met. In our game, meeting the target guarantees payout, but payout is already associated with sacrifices compared to the status quo (i.e. the initial endowment), as in the COP negotiations. Additionally, players in our asymmetric treatments received heterogeneous endowments, whereas those in (3) received symmetric endowments and a subset of players were ‘forced’ to contribute to a climate fund in the first three rounds to create asymmetry for the following seven rounds. [↑](#footnote-ref-4)
